# Shaping Development Through Parents: A Scalable Approach to Early Learning

**DOI:** 10.1111/desc.70279

**Published:** 2026-09-04

**Authors:** Gonzalez‐Gomez Nayeli, Naso Cecilia Eugenia, Tierney Fiona, Soba Roxana, Vazquez Claudia, Hendry Alexandra

**Affiliations:** ^1^ The Centre for Psychological Research Oxford Brookes University Oxford UK; ^2^ Hospital Aleman de Buenos Aires Buenos Aires Argentina; ^3^ Inter‐American Development Bank Washington USA; ^4^ Centre for Emerging Minds Research Department of Experimental Psychology University of Oxford Oxford UK; ^5^ Centre for Developmental Science School of Psychology University of Birmingham Birmingham UK

**Keywords:** home learning environment, parenting, parent‐child activities, SDG, socio‐economic status

## Abstract

Early childhood is a sensitive period for cognitive, language, and socio‐emotional development, with parental engagement playing a central role. In Argentina, families facing socioeconomic adversity often encounter barriers that limit access to developmentally enriching parent–child activities; heightening developmental risk amid ongoing economic instability. We conducted an exploratory proof‐of‐concept study adapting low‐cost parent–child activity packs—originally co‐developed in the UK—for use in Argentina. The adaptation prioritised cultural relevance, developmental suitability, material accessibility, and day‐to‐day feasibility, with guidance from a local healthcare professional to ensure alignment with local caregiving practices. The adapted packs were distributed to 73 vulnerable families who attended one of eighteen Early Childhood Centres (CPI *Centro de Primera Infancia*). Parents provided feedback through surveys (*N* = 73) and sessions (*N* = 45). Infant development (*N* = 73) was assessed with the Bayley‐III Scales. Parents reported increases in the frequency and variety of parent–child activities, alongside significant improvements in indicators of home learning environment quality. Thematic analysis identified perceived benefits for children (enjoyment, stimulation), families (stronger emotional bonds), and caregivers (greater capability, opportunity and motivation to engage in parent‐child activities), as well as suggestions for refinement. As expected in a short‐term, small‐scale study of a light‐touch programme, no significant changes were observed in Bayley‐III scores. As an exploratory proof of concept, the results indicate that culturally‐adapted, low‐cost interventions are acceptable and can help strengthen caregiver support and early learning in disadvantaged contexts. Aligned with SDGs 1,3,4, and 10, this model offers a scalable approach to enhancing parental engagement and equitable access to early learning.

## Summary

- Co‐produced activity packs increased parent‐child engagement in vulnerable Argentinian families.
- Parents reported enhanced capability, opportunity, and motivation to engage in developmentally supportive play.
- Despite no short‐term developmental gains on Bayley‐III, parents perceived improvements in children's communication, motor, and socio‐emotional skills.
- ‘Playful Packs’ offer a low‐cost, scalable complement to parenting programs, supporting SDG 4.2.1 by improving early learning access in LMICs.

## 1 Introduction

The first three years of life are fundamental to individual and societal outcomes later in life (Britto et al. 2017; Richter et al. 2017). Early skills in social‐emotional, cognitive, motor and language domains are implicated in a cascade of effects on outcomes as diverse as educational attainment, income, recourse to welfare assistance, involvement in the judiciary system, and physical health (Ahmed et al. 2021; Belfield et al. 2006; Moffitt et al. 2011). Thus, supporting early child development is key to achieving the Sustainable Development Goals about education (United Nations 2015, SDG 4.2.1), and more broadly to ‘ensure that all human beings can fulfil their potential in dignity and equality’ (SDG 1).

In turn, children's early skills are shaped by multiple interacting factors. Dynamic systems theory highlights the complex, reciprocal influences among biological (including epigenetic), psychological, behavioural, and social processes (Cantor et al. 2021). These processes unfold through interactions between the macro‐level (e.g., societal structures) and micro‐level (e.g., family relationships) contexts (Bronfenbrenner and Morris 2007). Within the micro context, nurturing care from caregivers—typically parents—plays a particularly influential role in early development (Britto et al. 2017). Although the impacts of variation within the micro context of the parent‐child relationship may not be readily observable in the short term, over the course of childhood, early differences in parent–child interactions and home learning environments accumulate over time to shape language skills, executive functions, and learning habits, which compound across childhood (Bush et al. 2020; Sulik et al. 2015).

### 1.1 The Critical Role of Parent‐Child Activities

Parent‐child activities provide an opportunity for multiple important components of nurturing care to intersect, such as opportunities for cognitive enrichment, sensitivity to children's physical and emotional needs, affectionate and responsive interactions, and a stable and somewhat predictable environment (Britto et al. 2017). Each of these components plays an important role in development: Cognitive enrichment during toddlerhood, through the provision of age‐appropriate resources and playful activities, is positively associated with child executive functions (Rosen et al. 2020; Valcan et al. 2018) and language ability (Tamis‐LeMonda et al. 2019). In fact, the UN SDG 4 recognises that ‘*Early stimulation increases duration of schooling, school performance, and adult income’*. Furthermore, affectionate and responsive interactions are important for social‐emotional development and cognitive development in general (Martini et al. 2024), and executive functions, in particular (Valcan et al. 2018). Thus, engagement in parent‐child activities, which can range from arts and crafts, to song and story sharing, physical games and sports, is positively associated with a range of child outcomes, at least in High Income Western societies, where the majority of this research has been conducted (Rakesh et al. 2024; Cates et al. 2023; Hendry et al. 2023; Hendry et al. 2022).

Whilst all parents can, in principle, support their child's development through engaging with them in enriching activities, they differ in the extent to which they do so (Bornstein et al. 2015; Bornstein and Putnick 2016; Hendry et al. 2022). The COM‐B model, developed by Michie and colleagues (Michie et al. 2011), offers a useful insight into why this may be by proposing that behaviour occurs through the interaction of three essential conditions: Capability, Opportunity, and Motivation. Capability refers to an individual's psychological and physical capacity to perform a behaviour. Opportunity captures external social and environmental factors that enable or constrain behaviour. Motivation encompasses reflective and habitual processes that direct behaviour. In the context of this study, barriers to regularly engaging in parent‐child activities can include lack of ideas, low confidence and positive experiences of play (‘capability’), time constraints and lack of access to appropriate resources (‘opportunity’), low interest in engaging in particular kinds of activities, and lack of awareness of the importance of parent‐child engagement in activities in the early years (‘motivation’; Gonzalez‐Gomez et al. 2025; Foulds 2023).

The Playful Packs project was developed in the UK using a co‐design approach involving parents and early years practitioners, to address common barriers to cognitively enriching parent–child interactions, particularly in socio‐economically disadvantaged contexts (Gonzalez‐Gomez et al. 2025). In doing so we aimed to increase the extent to which children were exposed to cognitively enriching age‐appropriate resources and playful activities, and to affectionate and responsive interactions—both during the study period and beyond. Workshops with parents and practitioners were used to identify everyday activities that support development, as well as practical and psychological barriers that limit engagement, drawing on the COM‐B model described above. These insights directly informed the design of the resources.

The resulting packs comprise simple, play‐based activities requiring minimal preparation, accompanied by low‐cost materials and brief guidance explaining how and why each activity supports children's cognitive, physical, and socio‐emotional development. By embedding developmental principles within everyday play, with a range of simple and enjoyable (for both parent and child) activities to choose from, the project addresses limitations of more resource‐intensive or prescriptive early stimulation programmes, while explicitly aiming to increase caregivers’ confidence, knowledge, and sense of agency.

In a UK evaluation, the Playful Packs were shown to be acceptable and feasible for families and were associated with positive changes in parents’ knowledge, attitudes, and reported practices (Gonzalez‐Gomez et al. 2025). The project's low‐cost, flexible, and caregiver‐mediated design makes it well‐suited for scaling up and for adaptation to other contexts, including Low‐ and Middle‐Income Countries (LMICs), where home‐based interventions have strong potential to support early development.

### 1.2 Supporting Early Child Development in Low and Middle‐Income Countries (LMIC)

Interventions to support early child development in LMIC are particularly important because poverty is a major risk factor for poor early developmental outcomes (Lu et al. 2016). To date, most parent‐mediated interventions to support early child development in LMIC are focused on parent education, advice or coaching (Ahun et al. 2023; Jeong et al. 2021; Tuñón et al. 2024). Whilst valuable, these approaches do not tend to address opportunity‐related barriers to parent‐child activities in LMIC, such as lack of access to physical resources, meaning that an important and potentially modifiable aspect of nurturing care has been overlooked. Moreover, education and advice programmes often aim for a broad reach in terms of the numbers of families supported, and the age of the children supported (Lachman et al. 2024; WHO 2012)—this affords the possibility of providing support at scale, but does not provide targeted insight into developmental mechanisms of change in early development in particular, or how to support those mechanisms. Where interventions have addressed opportunity‐related barriers to parent‐child activities by providing tangible age‐appropriate resources targeted to the toddler period, they have done so alongside parent coaching led by practitioners (e.g., community health worker or nurse; Abessa et al. 2019; Abimpaye et al. 2020; Attanasio et al. 2014; Chang et al. 2015; Gardner et al. 2005; Grantham‐McGregor et al. 2020; Hartinger et al. 2017; Madden et al. 1984), making it difficult to deduce whether or not resource provision is in itself an important factor in supporting child outcomes via increases in parent‐child activities.

This study contributes to addressing a key evidence gap by examining the adaptation of a caregiver‐mediated early stimulation intervention to a Low‐ and Middle‐Income Country (LMIC) context, focusing on Argentina. In Argentina, the population is highly concentrated in cities, and the country has experienced sustained economic instability, with annual inflation exceeding 100% and over 40% of the population living below the poverty line (INDEC, 2023). Like many children in LMICs, on average, young children in Argentina have limited access to learning resources in the home (Vegas et al. 2024), and socioeconomic and regional inequalities in caregiving and early development persist. Large international surveys further indicate that, even in relatively high‐performing LMICs such as Argentina, substantial proportions of caregivers do not engage regularly in cognitively stimulating activities with young children, particularly in lower‐income families (Cuartas et al. 2020). Some studies have found high levels of developmental disorders among vulnerable young children in the country (Carlos‐Oliva et al. 2020).

Although nationally representative estimates of developmental delay for children under three years of age are not available, converging evidence from developmental screening studies and national survey data suggests that by ages three to four, approximately 10%–30% of Argentinian children show suspected developmental concerns (Garibotti et al. 2013; Vegas et al. 2024), with higher risk among children from socio‐economically disadvantaged households. Whilst genetic and epigenetic factors are a key causal factor in developmental delays, psychosocial factors play an additional role in the increased rates observed within socio‐economically disadvantaged populations (Cattan et al. 2024; Cantor et al. 2021) and are a tractable target for change through early intervention. Children showing developmental delays within the first three years of life are at particular risk of poor outcomes (Shevell et al. 2005) yet often lack access to early intervention services. These patterns highlight the need for low‐cost, scalable approaches that support caregiver‐mediated cognitive stimulation in the home. Thus, in this study we focus on Argentinian children already identified as at risk of developmental delays. We present findings from an exploratory, proof‐of‐concept pilot study examining the feasibility, acceptability, and initial promise of adapting a set of resource packs—originally developed in the UK—for use with Argentinian families from vulnerable backgrounds. The packs were designed to shift everyday behaviours relating to parent‐child engagement in enriching activities as a proximal mechanism that may support optimal long‑term developmental trajectories through increasing child exposure to cognitively enriching age‐appropriate resources and playful activities, and to affectionate and responsive interactions. We aimed to achieve this shift through increasing parents’ capability, opportunity and motivation to regularly engage in a range of activities with their child. Our primary aim therefore was to assess acceptability, feasibility, and parent‑reported changes in parent‐child engagement, triangulated across multiple methods (questionnaires, informal one‑to‑one and group discussions, and creative participatory tasks). We did not anticipate that a light‐touch intervention would lead to observable changes in children's developmental ability within the short five‐month period of the current project. Nevertheless, to provide further contextual information we monitored children's developmental abilities prior to and after access to the packs.

## 2 Methods

### 2.1 Study Overview

The present report focuses on the adaptation and evaluation of ‘Playful Packs’—*Jugamos y Aprendemos* (‘We play and we learn’). Playful Packs formed one arm of a broader intervention project with children identified as at risk of developmental delay (according to baseline Bayley‐III Scales assessments). In the larger project, a separate group of families (*N* = 73; not included in this paper) received ‘First Steps’ (Vazquez and Naso 2025), a cultural adaptation of the Reach Up and Learn programme (Walker et al. 2018), which did directly target child development. The current paper reports exclusively on the Playful Packs group (*N* = 73). As part of the wider study protocol, infants in this group were assessed using the Bayley Scales at baseline, parents received the activity packs with brief practitioner guidance, and children were reassessed approximately five months later (M = 5.3 months, SD = 0.6; *N* = 62). Parents also completed a questionnaire on their perceptions and beliefs (*N* = 73), and a subset (*N* = 45) took part in three feedback sessions to discuss their experiences.

Because local practitioners advised that families in this vulnerable population might feel uncomfortable in formal interview settings, we provided multiple, flexible ways for parents to share their experiences, as capturing changes in parental perceptions, knowledge, and behaviour was the primary outcome of the activity packs. These changes represented the core targets of ‘Playful Packs’, and thus gathering rich, accessible, and culturally sensitive feedback was essential. To support this, we offered questionnaires, informal one‑to‑one conversations, informal group discussions, and several creative or anonymous formats such as the jar task, post‐it notes, and a ‘can‑recorder’ inspired by the string‑telephone game. These options were offered to reduce pressure and allow parents to respond in whichever format felt safest. In practice, parents were highly open and willing to engage during the facilitated discussions, and very few used the anonymous or creative formats. Nonetheless, ensuring a range of response options was an important step for inclusivity and for capturing our main programme outcomes as accurately and comfortably as possible.

### 2.2 Participants

73 infants aged between 4 and 21 months (Mage = 13.99m, SD = 4.56; 38 males and 35 females) and their caregivers took part in this study; see Table 1 for caregiver demographics. All infants attended one of eighteen Early Childhood Centres (CPIs) located mainly in the southern area of the City of Buenos Aires (see SM1 for full list), and in districts with the highest rates of social vulnerability and infant mortality. Access to CPIs is based on the Social Vulnerability Index (SVI), which scores neighbourhoods based on factors like poverty, homelessness, access to services, housing, health, education, employment, and demographics. Higher scores indicate greater vulnerability; the overall SVI score ranges from 0 (least vulnerable) to 1 (most vulnerable; see SM2 for more details). Children were eligible for the study if they were between 4 and 24 months old, attended a CPI, and showed signs of developmental delay at the time of evaluation. The term developmental delay refers to children who exhibit slower acquisition of developmental milestones but do not meet diagnostic criteria for a developmental disorder. For this study, and based on psychometric criteria, developmental delay was defined as scoring 5, 6, or 7 on one or more domains of the Bayley‐III. Children who scored 8 or higher (average range) in all domains were excluded, as were those with a history of congenital malformations or diagnosed sensory or developmental disabilities.

**TABLE 1:** Caregivers’ education level.

Education level	*n*	*%*	Cumulative %
Incomplete primary education	2	2.74	2.74
Completed primary education	6	8.22	10.96
Incomplete secondary education	15	20.55	31.51
Completed secondary education	27	36.99	68.50
Incomplete tertiary/university education	10	13.70	82.20
Completed tertiary/university education	13	17.81	100

Out of the 73 families who completed the assessments, 58 of the respondents were the mother of the child, 13 were the father and 2 others (i.e., grandparents). In Argentina, mandatory education spans 14 years: two years of early childhood education (ages 4 and 5), followed by 6 or 7 years of primary and 5 or 6 years of secondary education, depending on the jurisdiction. Since 2014, this full 14‐year cycle has been compulsory nationwide. Tertiary and university education are not mandatory. In our sample, most caregivers had secondary education or below (68.49%), equivalent to high school (see Table 1 for details). Importantly, 31.51% of caregivers in our sample had not completed mandatory education, similar to the national average of 32.6% (United Nations Educational, Scientific and Cultural Organization [UNESCO], 2025).

A total of 62 infants completed the Bayley‑III at both baseline and end‑point. For the remaining 11 children who did not receive the follow‑up assessment, four attended a centre that was closed during the study period due to infrastructure problems, four stopped attending their centres altogether, and three were absent on the days when assessments were conducted (see Discussion section on logistical challenges for further details).

This study received ethics approval from the Independent Ethics Committee of Hospital Alemán (CEIHA), validated by the Central Ethics Committee of the Buenos Aires Ministry of Health (CCE), and registered in the Buenos Aires Health Research Registry Platform (PRIISA.BA; registration code 12279). All procedures performed in this manuscript were in accordance with the 1964 Helsinki Declaration and its later amendments or comparable ethical standards. All participating caregivers provided informed consent on behalf of themselves and their child.

### 2.3 Activity Packs and Adaptation

Building on prior work identifying common barriers to parent–child engagement in the UK, the research team co‐developed three activity packs with parents and early years practitioners, designed specifically to address these challenges. Each pack included tangible materials (e.g., paints, toy animals), a companion storybook, and 10 simple, play‐based activity ideas—freely available online (https://tinyurl.com/5yapnx66). Activities were designed to be fun, easy to set up and clean up (within 5 minutes), and focused on play rather than outcomes. Easy‐read activity cards offered suggestions for adapting and extending each activity to suit children's interests and developmental levels (see Gonzalez‐Gomez et al. 2025 for details).

For use in Argentina, the packs were translated into Rioplatense Spanish and culturally adapted in collaboration with a local early childhood healthcare practitioner, who has extensive experience working with families across the socio‐economic spectrum and in‐depth knowledge of the context of vulnerable families participating in our study. Some UK activities were deemed unsuitable for the Argentinian context—for example, outdoor exploration activities like bug hunts were excluded due to safety concerns related to environmental contamination. The adaptation process was conducted activity by activity, considering multiple factors: the relevance of each activity to the local context, the suitability of materials, the clarity and simplicity of instructions, the language used, the images used, and the appropriateness for the slightly younger age range of infants in the Argentinian sample (4–36 months, compared with 8–36 months in the UK). These considerations were particularly important given that some caregivers may have limited familiarity with play‐based developmental interactions and that literacy levels may vary across families. Accordingly, the packs were designed to minimise reliance on written instructions, use simple and accessible language, and provide flexible, open‐ended activities that do not require prior experience with play‐based approaches.

**FIGURE 1:**
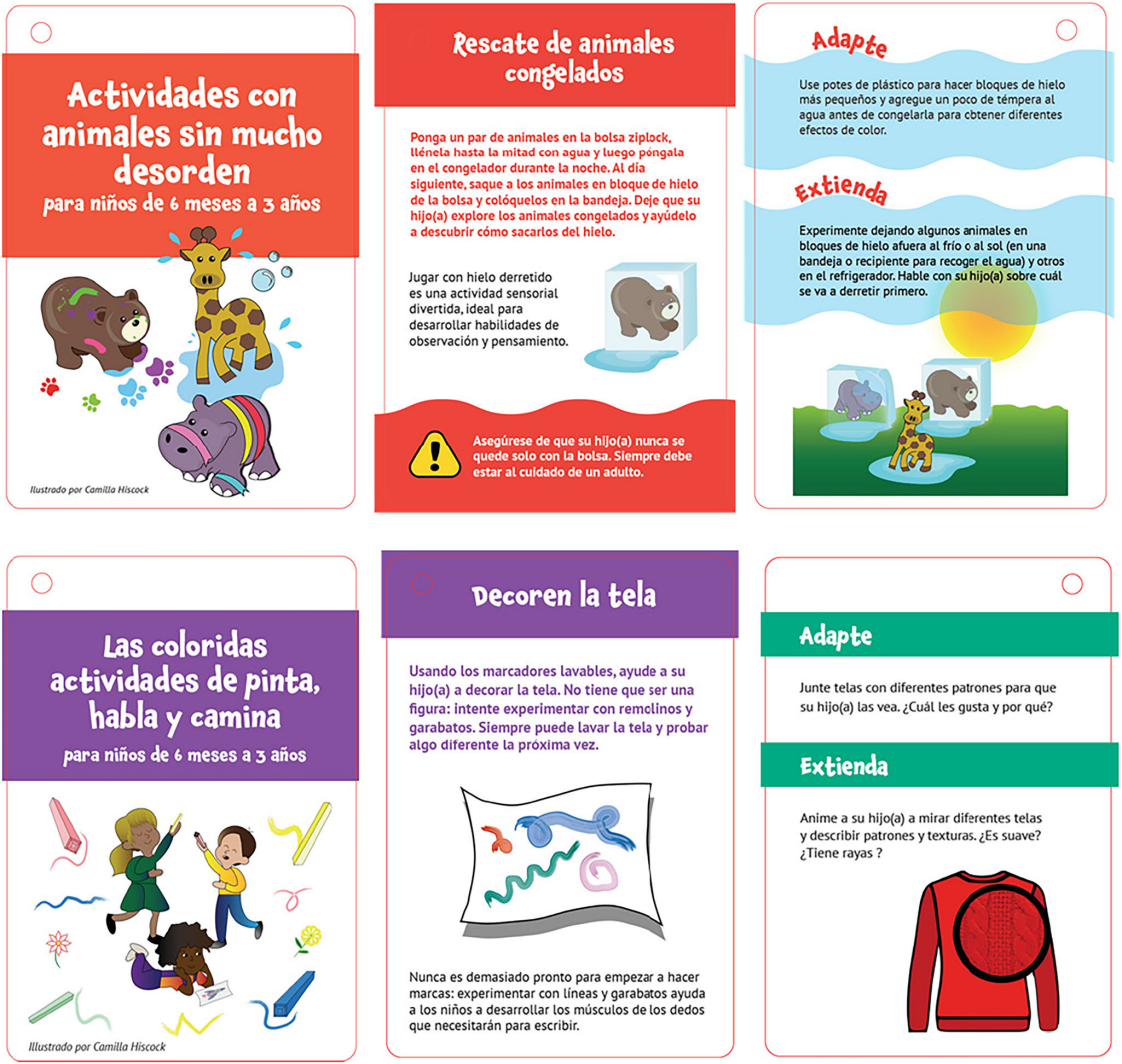
Example activities from animal activities without much mess (top panel) and the colourful paint, talk, and walk pack (bottom panel).

From the original 30 UK activities (10 per pack), 23 were selected for use in Argentina. One additional activity—peekaboo using a sheet of fabric—was added to better suit younger infants (4–6 months), as the Argentinian sample included children younger than the original target age range (8–36 months). The final set of 24 activities was organised into two adapted packs: Animal Activities Without Much Mess (*Actividades con animales sin mucho desorden*); and The Colourful Paint, Talk, and Walk Activities (*Las coloridas actividades de pinta, habla y camina*). Each pack included materials (e.g., paints, toy animals, tape), a storybook, and printed activity cards (see Figure 1 for examples and SM3 for a summary of Cross‐context Adaptation of Playful Packs).

### 2.4 Materials

#### 2.4.1 Parent Feedback Questionnaire

A custom questionnaire assessed the usability, perceived value, and impact of the activity box. It included Likert‐scale and multiple‐choice items across key domains: frequency of engagement, perceived usefulness, content appeal, instructional support, and parent‐perceived impacts. Parents rated items on 4‐ or 5‐point scales and responded to open‐ended questions. The questionnaire was completed on paper during the endpoint session, with evaluators available for clarification. (See SM4 for full questionnaire.)

#### 2.4.2 Family Care Indicators (FCI) Questionnaire (Frongillo et al. 2003)

To assess the quality of the home caregiving environment, the study used the Family Care Indicators (FCI) module from the UNICEF Multiple Indicator Cluster Surveys (MICS), developed in collaboration with UNESCO (Frongillo et al. 2003). The FCI is a standardized caregiver‐report tool designed to capture key aspects of early childhood stimulation and support in the home. The questionnaire includes items across several domains:

- Availability of Learning Materials: Presence of books, toys, and other educational resources in the home.
- Engagement in Stimulating Activities: Frequency with which caregivers engage in activities such as reading, storytelling, singing, playing, and naming/counting with the child.
- Support from Other Household Members: Involvement of other adults in the child's learning and play.
- Access to Early Childhood Programs: Participation in formal or informal early learning opportunities.

Responses to FCI items are categorical, for example, reporting the number of books or toys in the home (e.g., ‘none,’ ‘1–2,’ ‘3–5,’ ‘6 or more’) or the frequency of activities with the child over the past week (e.g., ‘none,’ ‘1–3 times,’ ‘4–6 times,’ ‘every day’), with higher scores reflecting greater levels of psychosocial stimulation. Although the FCI was developed internationally, it has been used in Argentina (UNICEF MICS, 2019), demonstrating its applicability to the local context. The FCI was administered during the baseline and endpoint. Caregivers completed the questionnaire in the presence of trained evaluators, who provided clarification as needed. Responses to individual FCI items were analysed separately, and an overall Engagement in Stimulating Activities score was also calculated by aggregating relevant items. Further details regarding the analyses conducted on these scores are provided in the Data Analysis section.

#### 2.4.3 Feedback Sessions

Families from three Early Childhood Centers (CPIs; i.e., C01, C02 and C03) were invited by the director of each CPI via their day‐to‐day communication channels to participate in feedback sessions facilitated by a member of the research team who had no prior contact with the families. This promoted an open and unbiased environment for sharing experiences. These centres were purposely selected to capture variation in levels and forms of socioeconomic vulnerability, including centres in high‐SVI areas and one representing urban families facing precarious employment conditions. One session was held at each of the three CPIs. The primary aim of each of these sessions was to gather qualitative feedback and explore families’ experiences with the activity packs. The sessions were intentionally informal to create a relaxed atmosphere and encourage open participation. Families were encouraged to talk about the following topics in group discussions or in one‐on‐one informal conversations with the facilitator (NGG):

1. how they had found the packs
2. what they found helpful or challenging
3. any changes they had noticed as a result of using the packs.

They could also share their thoughts by writing comments on sticky notes, or by participating in a ‘ feedback jar’ activity (described below) or responding to questions privately using a ‘can‑recorder’ inspired by the string‑telephone game. In practice, the can‑recorders were not used, as parents felt comfortable speaking openly during the feedback sessions. Families were invited, by their CPI, to take part in these sessions voluntarily, and they were organised around drop‑off and pick‑up hours to maximise participation; however, due to absenteeism, work commitments, and other logistical challenges, not all parents were able to attend. A total of 45 families took part in these sessions. In all three centres, the centre director was also present in the discussion and, in one centre, two teachers also chose to share their views as they had used the packs in their own practice (even though the packs were not originally intended for use in teaching).

**FIGURE 2:**
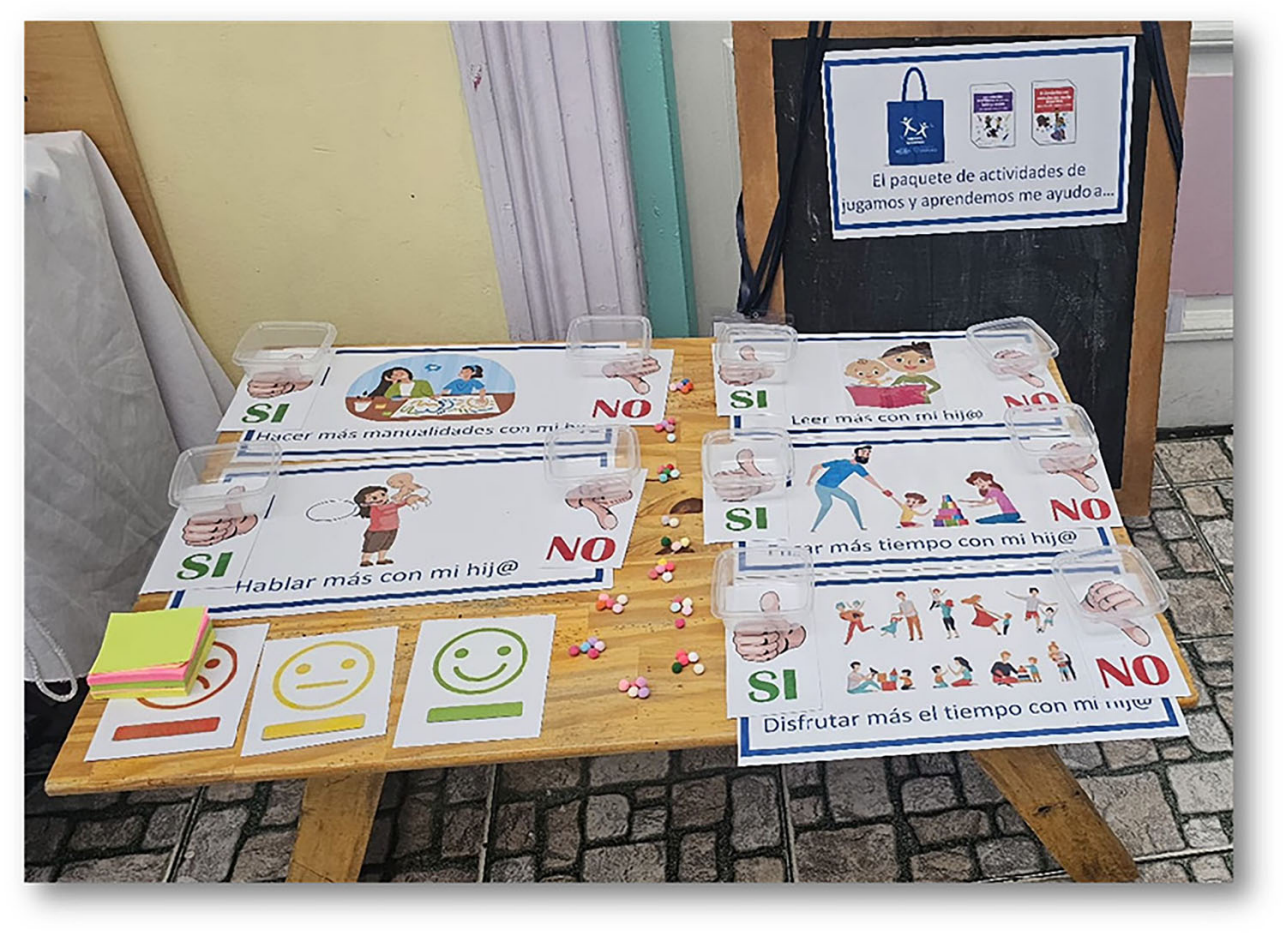
Example of jar task.

**Feedback Jar Task**. As part of the feedback sessions, families participated once in an interactive ‘feedback jar’ task designed to gather quick, accessible responses to five yes/no statements. Each participant received a set of pompoms and was asked to place them in jars to indicate their answers (see Figure 2). All statements began with the prompt: ‘The activity pack helped me…’ The five statements were: …spend more time with my child? …talk more with my child? …do more arts and crafts with my child? …read more with my child? …enjoy time with my child more? This method allowed families to provide feedback in a simple, engaging, and non‐verbal way, making it especially suitable for group settings and participants with varying literacy levels. Each Feedback Jar item was analysed independently. Frequencies and percentages of parents selecting ‘Yes’ and ‘No’ responses were calculated for each item and are presented in the Results section.

#### 2.4.4 Bayley Scales of Infant and Toddler Development, Spanish Version (Bayley‐III; Weiss et al. 2010)

The Bayley‐III is a standardised instrument designed to evaluate the development of infants and toddlers aged 1 to 42 months. It provides comprehensive information across five key domains: Cognitive, Language (Receptive and Expressive), Motor (Fine and Gross), Social‐Emotional, and Adaptive Behaviour. For this study, the Cognitive, Language, and Motor subscales were administered directly to the children by trained professionals certified in Bayley‐III administration. While there is no culturally adapted version of the Bayley for Argentina or other Latin American Spanish‐speaking countries, the Spanish version with Spanish norms is widely used in this context (Seibel et al. 2024). Notably, a study assessing the internal consistency of the Bayley III in an Argentinian sample during the first year of life reported good internal reliability across all age groups (Rodríguez et al. 2005). Each CPI provided a room with minimal distractions, equipped with a mat, a child‐sized table, and a chair to ensure an appropriate setting for the assessment. The Bayley‐III was administered twice, just before parents received the activity packs and a second time approximately five months later (M = 5.11 months SD = 0.69). Bayley‐III raw scores were converted into age‐normed percentile scores following standard administration guidelines, allowing children's performance to be evaluated relative to same‐age peers.

### 2.5 Data Analysis

Quantitative and qualitative data were analysed and reported separately, but the findings are integrated in the discussion section, which considers the changes brought about by ‘Playful Packs’.

#### 2.5.1 Quantitative Parent Feedback Questionnaire Data

Data were analysed using Jamovi version 2.3.28 (The Jamovi Project, 2025). All participants fully completed the survey, thus there were no missing data. Analyses of the Quantitative Parent Feedback Questionnaire were conducted using descriptive statistics only. Specifically, frequencies and percentages were calculated for each item.

#### 2.5.2 Qualitative Parent Feedback Questionnaire Data

To identify parent‐perceived changes due to ‘Playful Packs’, content analysis was applied to open‐text responses from the survey. FT^1^ initially coded the responses to identify common parent‐reported changes. Where parents had expressed multiple changes, multiple codes were assigned to that individual response. In discussion with NGG, initial codes were grouped into related concepts, and final codes were named (see SM5). Frequencies and percentages of participants expressing each code are reported.

#### 2.5.3 FCI Scores

Mean baseline and endpoint scores, as well as the corresponding differences, were calculated for each item and the overall Engagement in Stimulating Activities section. Wilcoxon signed‐rank tests were carried out to identify whether there were any significant changes in the home environment following pack introduction.

#### 2.5.4 Jar Task

Frequencies and percentages (to indicate proportions) of parents agreeing and disagreeing with each question are presented.

#### 2.5.5 Parent/Teacher Discussions and 1:1 Conversation Data

NGG transcribed and translated the feedback data from group discussions and 1:1 conversations from Spanish to English (See SM6 and SM7 for full transcripts in Spanish and English). Transcripts included data from 32 participants (22 mothers; 6 fathers; 2 centre directors and 2 teachers) who had made spoken contributions in discussions and 1:1 conversations during feedback sessions, in which 45 families, 2 centre directors and 2 teachers were present.

Reflexive thematic analysis (Braun and Clarke 2006, 2019) was applied to transcript data. This involved (1) familiarisation with the data through repeated reading of the dataset, (2) coding of the data, (3) grouping of codes into initial themes, (4) theme review, (5) naming and defining themes, and (6) writing up the analysis. This was an iterative process, which involved returning to transcripts at each stage to ensure participants’ experiences were captured as fully as possible. An inductive, data‐driven approach was taken to the analysis, and interpretation was at the semantic level, reflecting participants’ surface‐level rather than latent meanings.

FT conducted the analysis using NVivo 14 software. FT initially coded transcripts with the research question in mind: ‘How did parents and nursery centre staff experience the Playful Packs?’, then grouped codes into initial themes (See SM8 for examples of codes associated with each theme). Themes were discussed collaboratively with NGG and AH to ensure they reflected patterns in the data and aligned with the research question, and to allow for different perspectives on participants’ meanings to be shared. Further discussions were undertaken during the write‐up stage to consider whether illustrative quotations accurately represented the themes and participants’ experiences. For example, one participant expressed some guilt around not having played with her children before using the box; following discussion and review of transcripts, her overall positive feelings were also identified. Care was taken to include quotations from participants across all centres and roles (teachers, centre directors, mothers, and fathers). Participant characteristics are indicated in brackets after each quote (e.g., ‘C02, Mother 8’ refers to Participant 8 from Centre 2, a mother). Minor clarifications were added in square brackets where needed.

Exact frequencies of participants expressing each theme are not provided, as group dynamics means particular topics may have been raised in some discussions but not others. Counts of frequency in qualitative analysis may represent greater importance or significance or may indicate a greater willingness or ability to talk about a given topic (Vaismoradi et al. 2013). We have therefore used terms such as ‘some’ or ‘many’ to give an indication of the extent to which the theme or subtheme was represented in the dataset. All findings reflect participants’ perceptions and should be interpreted accordingly.

**Rigour and Reflexivity**. The research team comprised two Associate Professors in Psychology (NGG, AH), and a postdoctoral research assistant (FT), all of whom hold doctorates in child development and parenting. All researchers were female and parents; two were White British and one was Latino (non‐Argentinian). There were no pre‐existing relationships with participants, though participants were aware of NGG's research credentials and the study's purpose. The absence of an Argentinian researcher within the analytic team may have limited the interpretation of culturally specific meanings, expressions, or contextual nuances in participants’ accounts. To help address this, the team engaged in regular discussions with local partners throughout the analytical process to reflect on culturally situated meanings and interpretations. The team's professional expertise—particularly in parenting practices that support child development—may have influenced the analysis. To mitigate this, the primary analysis was conducted by FT, who was not involved in pack development or other aspects of the intervention. FT kept a reflexive journal throughout the analytical process, which she used to reflect on the team's reactions to the data and analytical decisions taken. Analytical discussions took care to prioritize participants’ surface‐level meanings and regularly referred back to transcripts to capture participants’ views and experiences as far as possible.

#### 2.5.6 Child Development (Bayley‐III) Data

Data from participants who had completed the assessment at both time‐points (*N* = 62) was analysed using Jamovi version 2.3.28 (The Jamovi Project 2025). Age‐normed percentile scores derived from the Bayley‐III were used in all analyses. Using percentile scores accounts for age‐related developmental differences; therefore, age was not included as a separate covariate in the analyses. Descriptive statistics (means and standard deviations) were calculated for baseline and endpoint scores. Paired‐sample *t*‐tests were used to examine changes over time in Cognitive, Language, and Motor scores following the introduction of the activity packs.

## 3 Results

### 3.1 Parent Feedback Questionnaire

#### 3.1.1 Engagement Frequency and Perceived Usefulness of the Packs

All parents (*N* = 73) reported engaging with the packs, with 46 (63.01%) reporting that they did so daily or almost every day (see Table 2). All parents considered the packs useful to some extent. 67 (91.78%) parents highly rated (4 or 5) the packs’ usefulness for giving them new ideas for activities, and 65 (89.04%) highly rated their help in understanding how such activities supported their child's development (see Table 2).

**TABLE 2:** Frequency of parent survey‐reported engagement level and perceived usefulness of ‘Playful Packs’ (*N* = 73).

Survey item	Parental response *n* (%)				
**Parental engagement**	Every day	Almost every day	Occasionally	Almost never	Never
Frequency of doing the activities with your child	7 (9.59%)	39 (53.42%)	27 (36.99%)	0 (0.00%)	0 (0.00%)
**Perceived usefulness of packs…**	5 (Very useful)	4	3	2	1 (Not useful at all)
…for giving new ideas	41 (56.16%)	26 (35.62%)	4 (5.48%)	2 (2.74%)	0 (0.00%)
…for helping understand child development	43 (58.90%)	22 (30.14%)	8 (10.96%)	0 (0.00%)	0 (0.00%)

#### 3.1.2 Instructional Support

52 parents (71.23%) found the cards with activity descriptions very useful for using the materials in the box, and 21 found them a little bit useful (28.76%). No parents reported that the cards were not useful at all.

#### 3.1.3 Content Evaluation

71 parents (97.26%) showed high levels of agreement (Agreed or Completely agreed) that the Playful Pack was appealing, 65 (89.04%) thought it was appropriate for their child's age, and 71 (97.26%) felt that it included fun activity ideas (see Table 5).

#### 3.1.4 Parent‐Perceived Impacts

69 parents (94.52%) agreed that they had spent more time doing activities with their children as a result of the packs, although four parents completely disagreed (5.48%; see Table 3). 71 parents (97.26%) agreed that packs had expanded the range of activities they now did with their child. 67 parents (91.78%) felt the packs had increased their confidence in supporting their child's development and had noticed improvement in their child's development as a result of the packs.

**TABLE 3:** Survey‐reported parental perception of content evaluation and impact (*N* = 73).

Survey item	Item response			
	Completely agree	Agree	Disagree	Completely disagree
**Content evaluation**				
The activity box is appealing.	38 (52.05%)	33 (45.21%)	1 (1.37%)	1 (1.37%)
The activity box is appropriate for my child's age.	27 (36.99%)	38 (52.05%)	7 (9.59%)	1 (1.37%)
The activity box includes fun activity ideas.	34 (46.58%)	37 (50.68%)	2 (2.74%)	0 (0.00%)
**Parent‐perceived impact**				
Encouraged more activity time with child	53 (72.60%)	16 (21.92%)	0 (0.00%)	4 (5.48%)
Expanded activity variety	47 (64.38%)	24 (32.88%)	1 (1.37%)	1 (1.37%)
Increased confidence in supporting child's development	48 (65.75%)	19 (26.03%)	4 (5.48%)	2 (2.74%)
Noticed improvements in child's development	45 (61.64%)	22 (30.14%)	6 (8.22%)	0 (0.00%)

#### 3.1.5 Overall Perceived Impact

Overall, 51 parents (69.86%) agreed with the statement that the packs had changed their thinking or made a difference for them; however, 22 parents (30.13%) did not. The changes they indicated in open‐text responses are presented in Table 4.

**TABLE 4:** Frequencies of parent‐reported changes brought about by the activity packs (*N* = 51).

Parent‐reported change due to Playful Pack	Count (%^*^)
Gave me ideas for more/new types of activities with my child	29 (56.86)
My child showed enjoyment and interest in the activities	16 (31.37)
Increased the time I spent connecting with my child through play	12 (23.53)
Made me aware of importance of spending time playing with child	10 (19.61)
Child made gains in knowledge/speech/motor skills	10 (19.61)
Changes in parent themselves, e.g., increased confidence	2 (3.92)

*Note*: ^*^Parents were able to indicate multiple changes, therefore, percentages do not add up to 100%.

### 3.2 FCI Scores

Parents (*N* = 58) indicated significant overall improvement in the quality of support for early development in the home from baseline following pack introduction (*p*<.001). Significant changes were found in relation to aspects supportive of language, literacy and mathematical knowledge (see Table 5).

**TABLE 5:** Differences in FCI scores at baseline and endpoint (*N* = 58).

*Questionnaire domains*	*Baseline*	*Endpoint*	*Diff*	*W(57)*	*p*	*Effect Size r*
*Reading*	*1.64*	*2.29*	*0.66*	*65.0*	*<.001*	−0.77
*Telling them stories or tales*	*1.69*	2.03	*0.35*	*119.5*	*0.016*	−0.49
*Singing songs*	*3.12*	*3.03*	*−0.09*	*307.0*	*0.632*	0.09
*Going for a walk*	*2.64*	*2.76*	*0.12*	*165.0*	*0.364*	−0.19
*Playing with their toys*	*3.22*	*3.31*	*0.09*	*114.5*	*0.467*	−0.17
*Drawing, painting, or scribbling*	*1.67*	*2.47*	*0.79*	*135.0*	*<0.001*	−0.69
*Playing at naming animals and their sounds*	*2.03*	*2.69*	*0.66*	*92.0*	*<0.001*	−0.67
*Playing at naming objects or colours*	*1.81*	*2.47*	*0.66*	*121.5*	*<0.001*	−0.64
*Playing at counting objects or saying numbers*	*1.90*	*2.24*	*0.35*	*213.5*	*0.054*	−0.36
*Talking during meals*	*2.83*	*3.12*	*0.29*	*151.5*	*0.091*	−0.35
*Inviting them to participate in household chores*	*1.91*	*2.53*	*0.62*	*192.0*	*0.003*	−0.53
*Overall*	*2.22*	*2.63*	*0.41*	*231*	*<0.001*	−*0.63*

Analyses were conducted using the Wilcoxon signed‐rank test given that the tests for divergence from normality were significant, this was expected given the ordinal, bounded nature of the FCI items.

### 3.3 Parent Feedback Sessions

#### 3.3.1 Jar Task

Parents had highly favourable perceptions of the benefits of the activity packs, with all indicating they had spent more time and read more with their children and had enjoyed doing so (see Table 6).

**TABLE 6:** Summary of parent‐reported benefits of the activity packs (jar task responses).

Centre	Spent more time with child		Talked more with child		Did more arts and crafts with child		Read more with child		Enjoyed time with child more	
	Yes	No	Yes	No	Yes	No	Yes	No	Yes	No
C01	11	0	7	4	10	0	18	0	10	0
C02	11	0	11	0	7	2	9	0	13	0
C03	9	0	9	0	10	0	7	0	11	0
*N*	31	0	27	4	27	2	34	0	34	0
(%)	(100.00)	(0.00)	(87.10)	(12.90)	(93.10)	(6.70)	(100.00)	(0.00)	(100.00)	(0.00)

#### 3.3.2 Parent/Teacher Discussions and 1:1 Conversations

Five themes were constructed from the parent and centre staff discussions and 1:1 conversations, one with associated subthemes. Four represent participants’ perceptions of the benefits of the packs and one outlines potential considerations for future development of activity packs (see Table 7).

**TABLE 7:** Themes and subthemes from parent and centre staff discussions and 1:1 conversations.

Themes	*Subthemes*
**A Children's enjoyment**	
**B Stronger family connections**	
**C Developmental gains**	
**D Support for caregivers**	*D.1 Upskilling parents*
	*D.2 Empowering parents and teachers*
	*D.3 Practical and emotional benefits for mothers*
**E—Future improvements**	

**(A) Children's Enjoyment**. Parents across the three centres, directors and teachers were overwhelmingly positive about ‘Playful Packs’, highlighting their children's enjoyment of engaging with the activity packs: *‘she really liked playing with the fabric. She loves when I cover her with it, and she also likes when I cover myself.’* (C02, Mother 3). There was variety in which activities children most enjoyed, with those mentioned including the ball, the animals, the paints and markers, the blanket/fabric, the ribbons, the ice‐making activity and the books. Mark‐making materials and books were often mentioned as popular new activities, which parents had not previously considered trying: ‘*The bag sparked her interest in books—now when she sees a book, she wants me to read it to her. We hadn't read to her before this.’* (C03, Mother 4). Parents and teachers appreciated the open‐ended nature of the materials, which allowed different ways of playing to continue to evolve and thereby sustain children's interest: ‘*He really likes trying new things—like all kids—so after a while he'd come up with new ways to use the same toys*.’ (C03, Father 10). Some parents reported that their children spent less time on screens as they found their new ways of playing more engaging.

Children's enthusiasm for the packs prompted them to ask for additional play, either verbally or non‐verbally. The portable nature of the bags facilitated even very young children to autonomously invite others to play with them: *‘We started incorporating it [playing with the pack] every day, until one day we forgot and the bag was hanging up—he went and showed us the bag to play. Now it's part of his routine—he wants the bag.’* (C02, Mother 6). In some families, their child's desire to play had helped shape new playtime routines. Children's enjoyment of the packs spread around the whole class for one teacher who tried the pack with a small group: *‘they kept asking for more—… And the rest asked me for it… they want to try it too.’* (C03, Teacher 1). Overall, children's enjoyment of the activities appeared to drive use of the packs across both home and nursery centre settings.

**(B) Stronger Family Connections**. Many parents valued the connection with their child that the activity packs brought about: ‘I really liked it, my wife did too. We don't have much time, but during the vacation, doing those activities with my daughter was really nice—I enjoyed it a lot.’ (C01, Father 6). Children's pleasure in activities, as described in the previous theme, brought about mutual enjoyment and feelings of closeness for parents: ‘when we did the blanket activity and made a little tent, he said ‘wow,’ and that was really special.’ (C01, Mother 2). Awareness of new details of children's interests, personalities and behaviours was often reported, such as ‘She's really sharp’ (C02, Mother 4). The director of one centre summed up the virtuous cycle of children's engagement in the activities and attentive parenting: ‘Kids who didn't speak at all now do, and the parents noticed the difference, which makes them even more engaged.’ (C01, Director 1). Parents and teachers also reported reciprocity between themselves and children in shaping activities: ‘We let them [children] be free and experiment, and they teach us too—we're here to learn… it's wonderful to watch them grow.’ (C03, Teacher 2). Broadly, participants expressed that the stimulation of the packs provided opportunities for them to pay attention and respond to their children's interests.

Parents valued not only the connection they experienced with their young child, but also the way the packs brought their whole family together. Some found that older siblings joined in with playing with younger children, sometimes for the first time: *‘It helped him communicate more with his siblings… they didn't really play with him before. Once they saw everything in the bag, they started playing games. He was thrilled.’* (C01, Mother 5). For some, the packs had enabled them to make the most of the scarce family time they had. Written materials in the packs emphasised the importance of parents spending time with their children, but many parents also experienced a felt sense of the importance of connection and emotional rewards of doing so: *‘The box helped me realise how important it is to share time as a family and how beautiful it is to be close and connected.’* (C02, Mother 8).

**(C) Perceived Developmental Gains**. The primary parent‐perceived benefit for child development was increased language and communication from their children. Messy play and creatively playing with animal toys and books were the activities most often described as encouraging speech: *‘Language is where he's the most delayed, and that's where he improved the most. He loved the book Lola and the Water… and always goes to bed with the book, pretending to read it. He struggles to make full sentences, but he started saying several words’* (C01, Mother 4). For children who were still pre‐verbal, some parents observed that their children had begun to communicate in other ways than speech*: ‘I think the activities sparked more curiosity in him. Now he goes around pointing at colours, he sees that there are different ones—I think it really helped him a lot.’* (C02, Mother 1).

An increase in mark‐making was also viewed by parents and nursery centre staff as benefiting children's development. Some noticed improvements in fine motor skills, some regarded it as *‘great stimulation’* (C01, Father 8) for the children and others valued the children's self‐expression through using paints and markers: *‘We started with pencils, crayons, and now the kids paint their folders by themselves—we just let them express themselves.’* (C03, Teacher 2).

Some parents also noticed their children showed increased self‐regulation, with their attention and/or behaviour improving since using the activity packs: ‘*He's more focused, sits still longer, and is calmer and more obedient.’* (C01, Mother 5).

**(D) Support for Caregivers**. There was a widespread feeling that the packs were supportive of parents themselves, by providing an enjoyable learning experience for them, as well as their children: *‘The box was a really lovely experience. We learned a lot as a family through the games—spending more time with our children and sharing learning moments together.’* (C02, Father 9). Three subthemes represent the main areas of support parents perceived.

***D.1 Upskilling Parents***. Many parents felt they had learned the importance of spending time with their children for their development: ‘Now with this, we realise how important it is… being there with him and having him learn that way. That's the progress we saw in him—and in ourselves. Before, I'd give him toys and go cook or do something else.’ (C02, Mother 6). Activity packs were widely valued for giving parents suggestions of how to play with and teach such young children: ‘Some activities may seem simple, but we don't think of them. It gave me new ways to play and simple activities that help my child.’ (C01, Mother 7). Some already played and spent time with their children, however the new ideas and suggestions were found to be helpful.

***D.2 Empowering Parents and Teachers***. Parents broadly felt empowered by being provided with the rationale behind activities and suggestions of adaptations: ‘So this was the guide I needed—and that many moms need… who want to give something to their kids but don't know how. I think this really helped them a lot—I see it in the other moms. Because you didn't just give out a box with ribbons and markers—you gave us the purpose behind it.’ (C02, Mother 10). Explanations of how activities could be used and their benefits for child development freed some parents to improvise and adapt in response to the needs of their individual children: ‘What happened to me was that I used to read stories just as they're written… But the guide we got said we could invent… That really helped my daughter.’ (C01, Father 8). This supported some to try new ways of playing when activities did not go as expected. One mother described how understanding the principles behind activities had unleashed her creativity in finding ways not only to use the provided resources, but also other materials she had to hand: ‘So I read, looked, and said, ‘Oh, I can do this too.’ For example, I took medicine bottles—like ibuprofen—washed them and filled them with lentils, little stones, and each made a different sound. Then I watched to see which sound she liked best.’ (C03, Mother 5). Centre directors particularly saw the packs as a ‘starting point’ (C03, Director 1), from which there were potentially limitless possibilities.

***D.3 Practical and Emotional Benefits for Mothers***. Activity packs helped some mothers practically by encouraging others in the family to play with their young children, in addition to themselves. In particular, fathers’ increased involvement was valued by a small number: *‘That's a big relief for us, too—it gives me a bit of time to do other things. I really think this is so important—so fathers are part of parenting too. Often, women carry all the weight because the men don't get involved.’* (C03, Mother 9). When siblings and fathers played with their young children, mothers perceived practical benefits of having more time for other tasks. Fathers’ involvement also had the emotional benefit of making some feel less alone.

Further to reducing emotional burdens of sole responsibility for childcare, some found the packs had supported their own emotional regulation in relation to parenting: *‘The bag really helped me—I found a new way to communicate with her. I used to feel very frustrated, I was overwhelmed, I didn't know how to teach her, and this gave me the tools to do that.’* (C03, Mother 2). For this mother, frustration was reduced as she learned new strategies, while another, who had been very anxious as a parent, found her confidence to let her son try new experiences increased.

**(E) Future Improvements**. Broadly, the activity packs were well‐received, however, there were some suggestions for improvement from parents and teachers, including adding more paints, as they ran out, and more books with different topics.

Although they were not designed for use with such young children, some parents used the activity packs with their babies. Some parents found this difficult to use the packs, particularly when babies wanted to put paint and other items in their mouths*: ‘we tried paper and paint, but she wanted to eat it’* (C03, Mother 5). Using books was also challenging for some parents, who perceived their young children as too active or unable to focus: *‘Books, not so much—he tends to throw them. So now I keep them in the closet until he's ready to pay more attention’* (C01, Mother 3). Some chose to wait to try activities until their child was older. However, one family creatively adapted activities to enable their baby to enjoy them. For example, instead of using paint, they used fruit for mark‐making: *‘she wanted to eat it [paint]. So my husband suggested trying fruit instead. He took her hand and said, ‘Look, (baby's name)’s hand,’ and so on. We also tried with orange and strawberry’* (C03, Mother 5).

Sibling jealousy and guilt around previous parenting was raised by one mother, who felt bad that she had not played in similar ways with her older children.: *‘I just keep asking myself—why didn't I do this with my older kids? I wasn't working, I had the time. That was my mistake—I admit it. I just didn't know. But it hurts now when they say, ‘Why didn't you do this with me?’ Now I'm trying to do something with each of them, to give them attention and be there for them’* (C03, Mother 8). While no other parents reported similar experiences, for this mother, using the pack with her baby had created conflict with her other children and negative emotions. Despite some challenging emotions, she felt positive about the pack for supporting her to spend quality time with her daughter and now spent time with each child *‘to give them attention and be there for them’* (C03, Mother 8).

### 3.4 Feasibility: Challenges and Implementation Issues

Given the feasibility‐focused nature of the present study, indicators of implementation, acceptability, engagement, and barriers to participation were considered primary study outcomes. Accordingly, these findings are presented in this section alongside other empirical outcomes.

In addition to overall acceptability, several feasibility‐related challenges emerged from parental reports and qualitative feedback. These included issues related to developmental appropriateness, material constraints, and family dynamics. To provide a clearer overview of feasibility, these challenges are summarised in Table 8.

**TABLE 8:** Feasibility of playful packs: Summary of challenges and implementation issues identified in the study.

Domain	Feasibility issue / challenge	Evidence from results	Illustrative example
**Child‐related factors**	Activities not always developmentally appropriate for youngest infants	Some parents reported difficulty using activities with babies (especially <6 months)	‘We tried paper and paint, but she wanted to eat it’ (C03, Mother 5)
	Limited attention span / readiness for certain activities	Books and some structured activities difficult for very young or highly active children	‘Books, not so much—he tends to throw them’ (C01, Mother 3)
**Material constraints**	Running out of consumable materials	Parents suggested need for replenishment (e.g., paints)	Requests for ‘more paints, as they ran out’
	Suitability/safety of materials for infants	Concerns about mouthing objects (e.g., paint) limited usability	Adaptations needed (e.g., using fruit instead of paint)
**Adaptation demands**	Need for caregiver improvisation and adaptation	Some families successfully adapted activities; others found it challenging	Example: replacing paint with fruit for safe mark‐making
**Family dynamics**	Sibling dynamics (jealousy, unequal attention)	One parent reported tension with older children	’Why didn't you do this with me?’ (C03, Mother 8)
	Emotional responses to parenting practices	Feelings of guilt about past parenting emerged for some	Same quote as above illustrates emotional burden
**Caregiver capability**	Initial lack of knowledge about how to play with young children	Parents valued guidance but indicated this was previously a barrier	‘Some activities may seem simple, but we don't think of them’ (C01, Mother 7)
**Engagement variability**	Variation in frequency of use across families, indicating differing capacity to integrate the packs into daily routines	While most parents engaged frequently, only 63% reported daily or almost daily use, and 36.99% reported using the packs only occasionally	7 parents (9.59%) used packs daily, 39 (53.42%) almost every day, and 27 (36.99%) only occasionally
**Implementation context (centres)**	Attendance and participation variability	Not directly in tabled results but evident in quantitative reporting of engagement across centres	Differences in engagement were noted descriptively
**Acceptability constraints (minor)**	Some activities not consistently used	Despite high overall engagement, certain components (e.g., books) were underused in some families	As above (books being stored until child is older)

In addition to these user‑ and context‑related challenges, feasibility also encompasses the financial and logistical viability of the intervention. The Playful Packs were designed as a low‑cost intervention, with an estimated cost of approximately £30 (∼$40) per pack, including materials such as paints, toy animals, books, and printed activity cards. However, in the present study, costs were influenced by contextual constraints, including limited local availability of materials, which required sourcing and transporting items internationally. These considerations highlight both the potential affordability and the practical challenges of implementation in low‑resource settings. Given the importance of cost‑effective approaches for expanding equitable access to early childhood learning opportunities, these findings are directly relevant to progress towards SDG 4.

### 3.5 Child Development

Assumptions of the *t*‐tests were evaluated using Shapiro‐Wilk tests and Q‐Q plots of the difference scores. The difference scores for Cognitive and Motor domains met normality assumptions (p = 0.052 and p = 0.270, respectively). Language difference scores showed slight deviations from normality (*p* < 0.001), with minor departures at the distribution tails, but the central portion was approximately normal (see Q‐Q plot, SM9). Given the sample size and approximate symmetry of the differences, paired *t*‐tests were considered appropriate.

Paired *t*‐tests comparing baseline and endpoint scores revealed no significant differences in Bayley‐III Cognitive, Language, or Motor percentile scores, although a marginal effect (p = .062) on cognitive percentile scores was observed (see Table 9; descriptive results by CPI are provided in SM10). As a sensitivity check, Wilcoxon signed‐rank tests were also conducted for the Language scores, which confirmed the same pattern of results.

**TABLE 9:** Summary of paired *t*‐test results for children's mean baseline and endpoint bayley‐III cognitive, language and motor percentile scores (*N* = 62).

Variable	Baseline percentile score m (SD)	Endpoint percentile score m (SD)	Difference m (SD)	t(61)	p	Effect size d
Cognitive	30.98 (18.91)	38.29 (24.31)	6.87 (29.82)	−1.90	.062	−0.24
Language	13.73 (10.42)	16.31 (14.51)	2.59 (15.34)	−1.32	.193	−0.14
Motor	29.32 (19.15)	30.36 (17.95)	1.03 (23.69)	−0.34	.735	−0.09

## 4 Discussion

This exploratory proof‐of‐concept study evaluated the adaptation and application of ‘Playful Packs’ (*Jugamos y Aprendemos*) in Argentina. The packs were designed as a low‐material‐cost (∼£30 per pack / ∼$40), minimally staff‑dependent, resource‐based approach to increase engagement in parent‐child activities by addressing barriers relating to capability, opportunity and motivation. We targeted parent‐child activities as a proximal caregiving process that may support children's long‐term development via increasing child exposure to cognitively enriching age‐appropriate resources and playful activities, and to affectionate and responsive interaction. Parent‐child interactions are a key influence on early development (Britto et al. 2017; Hendry et al. 2022; Hendry et al. 2023), and improving access to playful learning opportunities is essential to achieving equitable early education (UN 2015, SDG 4.2.1). Our aim was to examine the acceptability, feasibility, and parent‑ and practitioner‑perceived effects of the packs among families with young children identified as at risk of developmental delay.

Across multiple data sources, parents reported perceived improvements in engagement with their children, including more frequent shared activities (e.g., reading, mark‑making, arts and crafts) and more conversation, reported across the Parent feedback survey, jar task, parent/teacher discussions and informal conversations and the FCI questionnaire. Notably, parents expressed their enjoyment of these activities across the parent feedback survey, jar task and parent/teacher discussions and conversations. Parental increases in confidence in and awareness of the importance of playing with their children were captured in both the feedback discussions and the parent feedback survey. FCI questionnaire data indicated that household environments improved significantly, particularly in activities supporting literacy, mark‐making, numeracy, and family participation. Taken together, triangulated qualitative and quantitative evidence suggest a coherent and positive picture of parents perceived shifts in beliefs, knowledge, and everyday interaction patterns. Although parental knowledge of the value of such interactions varies across LMIC contexts (e.g., Foulds 2023; Gladstone et al. 2018; Hollowell et al. 2019), the present findings suggest that, in this Argentinian sample, parents reported increased awareness and understanding following their engagement with the packs.

### 4.1 Mechanisms of Change in Parenting Behaviour

Parents reported benefits in each of the dimensions within the COM‐B framework (Michie et al. 2011): In terms of capability, simple activities and brief guidance appeared to build ideas and confidence for symbolic, sensory, and literacy‑related play; this aligns with findings from the UK sample, where similar packs increased parents’ knowledge and confidence (Gonzalez‐Gomez et al. 2025). In terms of opportunity, ready‑to‑use materials and flexible suggestions helped families fit play into routines and involve siblings or fathers, within the constraints of time, resource, and safety risks frequently reported in LMIC contexts (Foulds 2023; Gladstone et al. 2018; Hollowell et al. 2019; Kim et al. 2021). In terms of motivation, shared enjoyment and a clearer understanding of play's value seemed to sustain engagement.

Together, these mechanisms appear to have increased both the quantity and quality of parent‐child engagement, despite the absence of facilitator‐led support or external coaching. In sensitively noticing and responding to their children's new‐found interests, parents reported becoming mutual partners in play with their children, which one centre director also highlighted. The new ways of playing, sensitive communication and, in some cases, new family playtime routines parents reported indicate that nurturing care, defined as including stimulation, responsiveness and safety (Britto et al. 2017), increased due to the activity packs. Nurturing care in the early years plays a critical role in development and is associated with numerous benefits, including later improved academic achievement and quality of life (Britto et al. 2017). For these reasons, increasing children's experience of nurturing care contributes to SDG1, allowing all to fulfil their potential.

Parental reports of feeling increased connection with their child align with parentally reported benefits of facilitator‐led parenting programmes internationally (e.g., Butler et al. 2020). The present study indicates that addressing barriers to engagement via resources and activity suggestions with explanations can bring about more responsive parenting in the absence of time‐ and resource‐intensive parenting programmes.

Importantly, the intervention was perceived as supportive and empowering, rather than prescriptive, by parents and nursery centre staff alike. This may explain the generally positive levels of reported engagement observed in the present study, particularly in contrast to more formal parenting programmes, which can sometimes engender parental resistance (Butler et al. 2020). Fear of judgement can be a barrier to participation in facilitated parenting programmes in LMICs (Ahun et al. 2023). As a non‐didactic, co‐produced, non‐facilitated intervention, ‘Playful Packs’ may help overcome such challenges. Parents in the present study reported engaging with a wide variety of activities their families enjoyed, indicating that flexibility in offering a range of play possibilities to suit families’ varied needs and interests is a key strength of the packs. This is in contrast to programmes which focus on one particular aspect of development, for example, reading, which may create pressure for parents (Nan & Tian 2025).

The packs also appeared to support more inclusive family engagement. Both mothers and fathers valued the opportunity to engage more with their children, and some mothers noted emotional and practical benefits from increased paternal involvement. This is important as maternal wellbeing is important for positive early childhood home environments (Gladstone et al. 2018; Koshy et al. 2021). By providing equal access to information and materials, the packs may help reduce inter‐parental conflict—a known barrier in mother‐centric interventions (Ahun et al. 2023)—and could complement facilitator‐led programs by increasing family‐wide acceptance of new parenting practices.

For some parents, accepting the way they were themselves parented can be an emotional challenge arising during parenting interventions (Ahun et al. 2023). This was not the case in the present study, although one mother reported the difficult emotions she now felt about her own past parenting and her older children's jealousy that she now played with their sibling but had not played with them. While she now makes time to engage with all her children, the potential for family conflict between siblings and for emotional distress around former parenting practices for some parents should be addressed in future interventions.

The present study also explored whether increasing parent‐child engagement was associated with developmental gains. Qualitatively, parents in the present study reported varied developmental changes in their children, including gains in motor, socio‐emotional and communication domains. The most widely reported were gains in speech and language, with parents describing their children's speech in response to symbolic and messy play. Many parents were also positively surprised by their young children's interest in reading and mark‐making. Parent perceptions from the present study concur to some extent with previous quantitative findings that parenting interventions promoting play in LMIC countries benefit fine motor skills (Abessa et al. 2019; Hartinger et al. 2017; Tuñón et al. 2024) and communication (Hartinger et al. 2017; Tuñón et al. 2024).

Despite generally positive reports of pack use and parental satisfaction, no significant short‐term improvements in child development were observed using the Bayley‐III Scales. Prior studies have shown developmental gains from parenting interventions in LMICs, such as improvements in cognitive and language skills in Uganda (Singla et al. 2015), social and motor development in Peru (Hartinger et al. 2017), fine motor skills in Ethiopia (Abessa et al. 2019), and communication and problem‐solving in Argentina (Tuñón et al. 2024). These differing results may stem from the more intensive nature of earlier interventions, often involving multiple home visits (Hartinger et al. 2017; Tuñón et al. 2024), or caregiver training sessions (Abessa et al. 2019; Singla et al. 2015), or additional care such as nutritional support (Abessa et al. 2019; Tuñón et al. 2024). Packs like those used here may be most effective as supplements to, or components of, practitioner‐led programmes. Additionally, participants in this study were already at risk of developmental delay, possibly requiring longer or more enriched interventions (Grantham‐McGregor et al. 1991, Grantham‐McGregor et al. 2020; Hartinger et al. 2017). Many studies detecting limited short‐term effects still report meaningful change over extended periods (Jeong et al. 2021; Van Aar et al. 2017), suggesting that the brief follow‐up period may have limited our ability to observe longer‐term developmental shifts.

Having established the packs' acceptability and feasibility, future research should consider including children with diverse developmental profiles at baseline and incorporating follow‐up assessments at least six months post‐endpoint. Long‐term tracking of parental engagement and child outcomes is essential, as short‐term gains from parenting interventions in LMICs often fade over time (Jeong et al. 2021). To determine the added value of resource packs beyond parent coaching, future studies should adopt a multi‐arm design, comparing coaching alone, coaching plus resources, resources alone, and a control group.

### 4.2 Generalisability to Other LMIC Contexts

While this study was conducted in Argentina, several aspects of the approach may be relevant to other low‐ and middle‐income country (LMIC) settings, particularly those facing similar structural constraints. The core design features of the Playful Packs—namely low‐cost materials, simple play‐based activities, and a non‐facilitated format targeting barriers to capability, opportunity, and motivation—address challenges commonly reported across LMICs, such as limited access to services, time constraints, and resource scarcity (Ahun et al. 2023; Jeong et al. 2021). These features align with global evidence highlighting the importance of caregiver–child interactions for early development (Britto et al. 2017). However, the findings should not be interpreted as directly generalisable to all LMIC contexts. Our results also underscore the importance of context‐specific adaptation, as implementation was shaped by local factors including material availability and cost, cultural beliefs about play and parenting, caregiver literacy levels, and broader socio‐economic conditions (Lansford, 2022). As demonstrated in this study, even within a middle‐income country, substantial adaptation was required to align the packs with families’ needs and realities. Therefore, while the underlying approach may provide a useful framework for supporting parent–child engagement in other LMIC settings, effective implementation will require iterative, locally grounded co‐production with caregivers and community stakeholders rather than direct transfer. Further research is needed to evaluate how such resource‐based interventions translate across diverse cultural and economic contexts.

### 4.3 Implementation Considerations

Logistical barriers are a key challenge in achieving equitable access to education for all in LMICs (SDG 4.2.1; Ahun et al. 2023). Notably, challenges in identifying and training delivery agents are key barriers for delivery of WHO CCD parenting programmes in LMICs (Ahun et al. 2023). Furthermore, time‐poor parents may be unable to attend group sessions and lack of infrastructure may make home visits unviable. ‘Playful packs’ are not facilitator‐led, yet delivered increased knowledge of the importance of early parent‐child engagement and parent‐reported behavioural change. This suggests that such resource packs could contribute to achieving the vital early stimulation on which later educational achievement is built (Britto et al. 2017) where logistical barriers make parental support otherwise impossible.

Nevertheless, the study also highlights the logistical challenges of implementing parenting interventions in middle‐income countries (MICs), especially among highly disadvantaged populations. Although initial uptake was strong, long‐term impact depends on sustainable resource supply chains and reliable distribution. Challenges included very high cost and low local availability of materials due to the economic crisis. For this reason, most materials had to be purchased in the UK, after which the packs were assembled and shipped to Argentina. This created additional logistical difficulties, including problems importing materials and the loss of one shipment in transit. In addition to these supply‑chain barriers, we faced substantial challenges due to high levels of centre absenteeism, which stemmed from structural vulnerabilities common in low‑resource urban settings. These had direct implications for data collection, often requiring sessions to be rescheduled multiple times or, in some cases, making it impossible to collect follow‑up assessments. Reasons for absences included work obligations, informal employment patterns, irregular childcare attendance, housing instability, and episodes of homelessness. Frequent residential moves often resulted in families changing the CPI they attended, while some households—particularly those living in refuges or experiencing severe material deprivation—struggled with day‑to‑day subsistence concerns that made long‑term planning difficult, which had an impact on their child's attendance to the CPI. Migration dynamics also played a role, with some families returning to their countries of origin, most commonly neighbouring countries such as Bolivia. Adverse weather further reduced attendance; in Buenos Aires, rainy days consistently resulted in high absenteeism across centres, especially in flood‑prone areas such as comunas 4 and 8, where eight CPIs are located. Infrastructure issues within the CPIs—such as power outages, flooding, and, in one case, the temporary closure of a centre due to a gas connection problem—also led to periodic suspension of activities. These factors illustrate the macro‐level processes that influence child development along with the micro‐level processes targeted within this project (Bronfenbrenner & Morris 2007). These interrelated contextual barriers also demonstrate the need for flexible, context‑sensitive delivery models to ensure equitable access and sustained engagement. Scaling such interventions in MICs will require addressing these logistical constraints through continued co‑production with parents, community organisations, and local service providers.

Beyond identifying these challenges, several lessons emerged that can inform future implementations. First, greater flexibility in scheduling—allowing sessions to be rescheduled and adapting meeting times to families’ availability—may help sustain engagement and support the gradual development of more stable routines. Second, offering a mix of in‑person and virtual sessions can help maintain participation when families are unable to attend due to weather, work demands, or housing instability. Third, group feedback sessions were especially valuable: families welcomed the opportunity to share their experiences and reflections on the activity packs, and these meetings strengthened relationships with staff and allowed practitioners to provide timely feedback on changes observed in the children. Together, these lessons highlight practical strategies to enhance feasibility and adherence in similar low‑resource contexts.

#### 4.3.1 Future Considerations

While participating parents generally reported high levels of satisfaction with the packs, suggestions for improvement included replenishing consumable materials and offering more activities suitable for babies, as families often used the packs with children of various ages. Partnership with community providers and ongoing collaboration is a further key component to be considered in future projects. The present project delivered packs via multiple centres and there appeared to be differences in engagement levels between centres, although small sample size precluded statistical analysis. Centre staff played a clear role in promoting pack use, highlighting the importance of community‐based delivery and trusted relationships for uptake (see Pellecchia et al. 2018, for a review).

### 4.4 Implications

Parent‐child activities are just one (important) facet of the child's developmental system. The activity packs do not—and were not intended to—address the broader challenges families face, and which also constrain children's development (Bronfenbrenner and Morris 2007; Cantor et al. 2021). Multi‐sector engagement and systemic change are also needed to address multi‐generational poverty across the globe (Cerf, 2023; National Academies of Sciences, Engineering, and Medicine 2024; Rahman and Pingali 2024). Within this broader context, our findings suggest that Playful Packs may be a promising low‑cost, adaptable complement to existing parenting and early‑learning initiatives, although this remains to be tested in larger and more diverse samples. While not a substitute for large‑scale programmes (e.g., WHO CCD; Ahun et al. 2023), the packs may help inform approaches to enhancing national efforts—such as Argentina's Integrated Early Childhood Development programme—by offering practical, accessible support for families facing economic and time constraints. Importantly, the model is flexible across levels of need: universally, it offers play‑based activity ideas with brief developmental explanations; for families experiencing hardship, it provides physical materials; and within more intensive interventions, it can serve as between‑session support, potentially increasing the ’dose' of parent–child engagement (Wallander et al. 2014).

More broadly, the approach aligns with the Nurturing Care Framework (NCF; UNICEF and WHO 2018), which positions the caregiver–child relationship as the central context for early development and defines nurturing care as providing stable environments that ensure health, nutrition, safety, early learning, and ‘responsive, emotionally supportive and developmentally enriching relationships’ (p. 1). The NCF is explicitly framed as a roadmap to the SDGs, particularly SDG 4 (quality early childhood development; Camarata et al. 2023). Within this framing, resource‑based supports that strengthen everyday playful learning at home may contribute to SDG 4.2 and, by improving reach among families facing time, cost, or literacy barriers, may also support SDG 10 (reduced inequalities) in access to early learning opportunities.

Playful Packs fit within a wider ecosystem of caregiver‑centred supports as a light‑touch, non‑didactic option. They do not replace multi‑session coaching or home visiting but can amplify such approaches by (i) providing ready‑to‑use materials and low‑demand activity ideas, (ii) reducing barriers linked to capability, opportunity, and motivation, and (iii) offering between‑session continuity that helps embed new habits at home. In Argentina, this enables the model to sit alongside existing services, helping extend reach where workforce capacity or family time constraints limit participation in more intensive programmes.

Overall, Playful Packs offers an accessible, flexible, and context‑sensitive model for supporting parents’ understanding of child development and strengthening everyday learning interactions in the home. This proof‑of‑concept work provides a foundation for future controlled evaluations to establish impact, cost‑effectiveness, and options for integration into Argentina's early childhood systems.

### 4.5 Strengths and Limitations

A key strength of this study is its mixed‑methods design, which enabled triangulation across multiple sources of evidence. Group discussions, individual feedback sessions, questionnaire data, the jar‑task measure, and the FCI together provided a richer understanding of how the activity packs were used and perceived and helped identify plausible mechanisms driving behaviour change. The FCI was intentionally used as a complementary rather than primary measure, as pre–post changes without a control group may reflect test–retest effects or typical developmental progression. Importantly, however, the FCI patterns were consistent with qualitative and quantitative feedback, strengthening confidence in the overall interpretation while acknowledging that causal attribution is not possible. The study also contributes novel evidence by focusing on a specific, culturally adaptable mechanism—parent–child activities—rather than more contextually‑bound constructs such as ‘responsive parenting.’

While the packs were co‑produced with parents and early‑years practitioners in the UK, they were subsequently adapted for the Argentinian context in collaboration with a local early‑years practitioner who reviewed the activities for cultural relevance, material accessibility, and appropriateness for the families served. Our findings suggest that further adaptation is still needed to ensure cultural relevance and avoid imposing normative parenting models. During implementation, it became clear that parents’ prior experiences, expectations, and understandings of play differed from those of parents in the UK. Several parents described having had little or no playful interaction in their own childhoods, and some were encountering the idea of play as a developmental tool for the first time. In addition, although written text was kept to a minimum, a subset of parents had low literacy levels, suggesting that text‑based explanations may not be the most accessible format. These observations highlight the importance of re‑adapting the materials—potentially through more visual or video‑based formats—and involving Argentine parents directly in a next stage of co‑production to ensure that the content reflects local beliefs, practices, and communication needs. Parenting practices are shaped by environmental and sociocultural contexts (Lansford, Gonzalez–Gomez Nayeli: conceptualization, investigation, funding acquisition, writing – original draft, methodology, validation, visualization, writing – review and editing, formal analysis, project administration, resources, supervision, data curation. 2022), and this study represents an initial step in an iterative, locally grounded process of adaptation and evaluation specific to Argentina.

Limitations include potential self‐selection bias, with under‐representation of less‐engaged families, whom future studies should aim to include along with more engaged families to better understand barriers to use. Participation in feedback activities was voluntary and not systematically tracked, which further limits the ability to assess the extent or representativeness of engagement in these qualitative components. In addition, implementation fidelity was assessed only through caregiver self‐report, with no independent measures (e.g., usage logs, follow‐up checks, or observational assessments of pack use), limiting the ability to verify frequency or quality of use. Although attempts were made to mitigate power dynamics, participants may have reported what they thought researchers wanted to hear. Group dynamics may have led participants to emphasise some aspects of the activity packs and underreport others. In addition, the presence of CPI directors during feedback sessions may have influenced participants’ responses by introducing additional power dynamics, potentially increasing social desirability bias or limiting the expression of critical views in group discussions. However, triangulation with survey feedback, the FCI questionnaire and the jar task indicates that the positive views captured in discussions were widely held. The small sample size, lack of a control group, and short study duration also limit conclusions about its effects on infants’ developmental outcomes. Given that children were identified as being at risk for developmental delay and therefore tended to score below normative means at baseline, any apparent movement toward normative scores over time should be interpreted cautiously, as such changes may partly reflect regression to the mean rather than effects of the activity packs.

## 5 Conclusion

This study shows that providing resource packs co‐produced with parents can increase parent‐child engagement in vulnerable families in Argentina without facilitator input. Parents broadly valued the approach and found the materials supportive, empowering and enjoyable. By addressing barriers to capability, opportunity, and motivation, ‘Playful Packs’ show promise as a practical, scalable complement to larger parenting initiatives, potentially furthering global progress towards equitable early education (SDG 1, 3, 4 and 10). Further research is needed to assess long‐term effects on infants’ developmental outcomes, understand barriers for less‐engaged families, and explore cost‐effectiveness and implementation at scale.

## Author Contributions

**Gonzalez‐Gomez Nayeli**: conceptualization, investigation, funding acquisition, writing – original draft, methodology, validation, visualization, writing – review and editing, formal analysis, project administration, resources, supervision, data curation. **Naso Cecilia Eugenia**: investigation, writing – review and editing, project administration, supervision. **Tierney Fiona**: writing – review and editing, writing – original draft, visualization, formal analysis, data curation. **Gonzalez–Soba Roxana**: investigation, supervision, writing – review and editing. **Vazquez Claudia**: investigation, writing – review and editing, supervision. **Hendry Alexandra**: conceptualization, investigation, writing – original draft, writing – review and editing, methodology, formal analysis, validation, visualization, resources.

## Conflicts of Interest

The authors declare no conflicts of interest.

## Supporting information




**Supporting Information**: desc70279‐supp‐0001‐SuppMat.docx

**Supporting Information**: desc70279‐supp‐0002‐SuppMat.docx

**Supporting Information**: desc70279‐supp‐0003‐SuppMat.docx

**Supporting Information**: desc70279‐supp‐0004‐SuppMat.docx

**Supporting Information**: desc70279‐supp‐0005‐SuppMat.docx

**Supporting Information**: desc70279‐supp‐0006‐SuppMat.docx

**Supporting Information**: desc70279‐supp‐0007‐SuppMat.docx

**Supporting Information**: desc70279‐supp‐0008‐SuppMat.docx

**Supporting Information**: desc70279‐supp‐0009‐SuppMat.docx

**Supporting Information**: desc70279‐supp‐0010‐SuppMat.docx

## Data Availability

The authors confirm that the data supporting the findings of this study are available within the article and its supplementary materials.

## Endnotes

1 FT, NGG, and AH correspond to the initials of the authors.
