# Supplementary material for: Shaping Development Through Parents: A Scalable Approach to Early Learning
Source: Dev Sci. 2026 Sep 4;29(6):e70279. doi: 10.1111/desc.70279 (PMC13543324; doi:10.1111/desc.70279)
Supplement: Supplementary file 1 [file DESC-29-e70279-s001.docx]

**Supplementary Material 1**

*Full List of Centros de Primera Infancia (CPIs; Early Childhood Centres)*

| CPI ID | Neighbourhood | Social Vulnerability Index |
| --- | --- | --- |
| C01 | Soldati | 0,377 |
| C02 | Balvanera | 0,165 |
| C03 | Soldati | 0,377 |
| C004 | Lugano | 0,307 |
| C05 | Belgrano | 0,098 |
| C06 | Retiro | 0,145 |
| C07 | Soldati | 0,377 |
| C08 | Constitución Mataderos | 0,206 |
| C09 | Flores | 0,19 |
| C10 | Flores | 0,19 |
| C11 | Barracas | 0,242 |
| C12 | Liniers | 0,18 |
| C13 | Mataderos | 0,219 |
| C14 | San Telmo | 0,175 |
| C15 | Colegiales | 0,111 |
| C16 | Mataderos | 0,219 |
| C18 | La Boca | 0,258 |
