# Supplementary material for: Shaping Development Through Parents: A Scalable Approach to Early Learning
Source: Dev Sci. 2026 Sep 4;29(6):e70279. doi: 10.1111/desc.70279 (PMC13543324; doi:10.1111/desc.70279)
Supplement: Supplementary file 2 [file DESC-29-e70279-s002.docx]

**Supplementary Material 4**

*Initial and Final Codes and Counts for Open-text Survey Responses Indicating Parent-perceived Changes due to ‘Playful Packs’*

| **Initial Codes** | **Counts** | **Final codes** | **Counts** |
| --- | --- | --- | --- |
| Gave ideas for more activities | 13 | Gave me ideas for more/new types of activities with my child | 29 |
| Introduced new types of activities with child | 14 |  |  |
| Gave ideas for activities with materials we already have | 2 |  |  |
| Made me aware of importance of spending time playing with child | 10 | Made me aware of importance of spending time playing with child | 10 |
| Child enjoys new activities | 7 | My child showed enjoyment and interest in the activities | 16 |
| Increased child's interest in books | 3 |  |  |
| Increased child's attention in activities | 3 |  |  |
| Increased child's interest in painting | 1 |  |  |
| Increased child's independence | 2 |  |  |
|  |  |  |  |
| Increased time spent playing with child | 9 | Increased the time I spent connecting with my child through play | 12 |
| Increased my connection with child | 3 |  |  |
|  |  |  |  |
| I changed a lot | 1 | Changes in parent themselves, e.g. increased confidence | 2 |
| My confidence increased | 1 |  |  |
|  |  |  |  |
| Child's speech improved | 5 | Child made gains in knowledge/speech/motor skills | 10 |
| Child's motor skills improved | 2 |  |  |
| Child learned | 3 |  |  |
