# Supplementary material for: Shaping Development Through Parents: A Scalable Approach to Early Learning
Source: Dev Sci. 2026 Sep 4;29(6):e70279. doi: 10.1111/desc.70279 (PMC13543324; doi:10.1111/desc.70279)
Supplement: Supplementary file 3 [file DESC-29-e70279-s003.docx]

**Supplementary Material 10**

*Bayley’s scores by Centro de Primera Infancia (CPIs; Early Childhood Centres)*

| Centre number | CPI Name | Sample size (Baseline/ Endpoint) | Cognitive  Baseline percentile score *m* (SD) | Cognitive Endpoint  percentile score *m* (SD) | Cognitive Difference *m* (SD) | Language  Baseline percentile score *m* (SD) | Language Endpoint  percentile score *m* (SD) | Language Difference *m* (SD) | Motor  Baseline percentile score *m* (SD) | Motor Endpoint  percentile score *m* (SD) | Motor Difference *m* (SD) |
| --- | --- | --- | --- | --- | --- | --- | --- | --- | --- | --- | --- |
| 1 | Alas de colores | N= 4 / 4 | 29.8 | 70.0 | 40.3 | 15.3 | 33.3 | 18.0 | 29.0 | 50.0 | 21.0 |
| 2 | Alicia en el país de las maravillas | N= 3 / 3 | 22.0 | 23.7 | 1.7 | 11.7 | 25.0 | 13.3 | 35.0 | 21.0 | -14.0 |
| 3 | Años felices | N= 5 / 5 | 25.0 | 48.0 | 23.0 | 19.2 | 17.2 | -2.0 | 15.2 | 31.6 | 16.4 |
| 4 | Bajitos de JD | N= 5 / 5 | 35.6 | 34.8 | -0.8 | 10.4 | 19.4 | 9.0 | 19.8 | 27.8 | 8.0 |
| 5 | Brotecitos | N= 3 / 3 | 46.0 | 30.3 | -15.7 | 7.3 | 10.4 | 3.1 | 37.3 | 40.3 | 3.0 |
| 6 | CAIPLI Norte | N= 6 / 2 | 20.5 | 20.5 | 0.0 | 15.5 | 3.5 | -12.0 | 59.0 | 24.0 | -35.0 |
| 7 | Casita San José | N= 3 / 2 | 43.5 | 50.0 | 6.5 | 20.5 | 25.0 | 4.5 | 46.0 | 24.0 | -22.0 |
| 8 | Casitas | N= 3 / 3 | 16.7 | 13.7 | -3.0 | 11.7 | 4.3 | -7.3 | 26.3 | 14.7 | -11.7 |
| 9 | Chonito | N= 6 / 5 | 55.6 | 18.4 | -37.2 | 26.6 | 16.2 | -10.4 | 37.2 | 20.0 | -17.2 |
| 10 | Dejando huellas | N= 4 / 4 | 35.0 | 28.8 | -6.3 | 13.8 | 11.5 | -2.3 | 46.0 | 29.5 | -16.5 |
| 11 | El soñado | N= 7 / 4 | 20.5 | 35.0 | 14.5 | 15.5 | 9.5 | -6.0 | 23.5 | 23.0 | -0.5 |
| 12 | Girasoles | N= 4 / 4 | 38.8 | 56.3 | 17.5 | 9.5 | 28.8 | 19.3 | 29.8 | 41.0 | 11.3 |
| 13 | La mona Jacinta | N= 2 / 2 | 43.5 | 69.0 | 25.5 | 6.0 | 12.0 | 6.0 | 46.0 | 47.5 | 1.5 |
| 14 | Mi casita | N= 4 / 4 | 30.3 | 54.5 | 24.3 | 13.3 | 18.0 | 4.8 | 13.0 | 38.8 | 25.8 |
| 15 | Mis primeros pasos | N= 3 / 2 | 7.0 | 50.0 | 43.0 | 6.0 | 14.5 | 8.5 | 34.0 | 54.5 | 20.5 |
| 16 | Raíces de amor | N= 3 / 3 | 22.0 | 35.0 | 13.0 | 18.7 | 21.3 | 2.7 | 19.7 | 22.0 | 2.3 |
| 17 | Rayuela | N= 5 / 5 | 24.2 | 33.8 | 9.6 | 6.2 | 7.8 | 1.6 | 20.8 | 20.8 | 0.0 |
| 18 | San Juan Evangelista | N= 3 / 2 | 26.5 | 17 | -9.5 | 13 | 5.5 | -7.5 | 31.5 | 29 | -2.5 |
|  |  |  |  |  |  |  |  |  |  |  |  |
