# Supplementary material for: Shaping Development Through Parents: A Scalable Approach to Early Learning
Source: Dev Sci. 2026 Sep 4;29(6):e70279. doi: 10.1111/desc.70279 (PMC13543324; doi:10.1111/desc.70279)
Supplement: Supplementary file 4 [file DESC-29-e70279-s004.docx]

**Supplementary Material 5**

**Transcript of Parent Feedback Sessions (Spanish)**

***C01***

Mamá 1

Porque me decoro la pared, porque mis le dicen ay gordo que hiciste, hiciste un Picasso. Hizo un círculo grande con unas rayitas así.

¿Qué actividades fueron las que más les gustaron?

Las pinturas, las temperas, aprendió a escribir su nombre, yo le hago dibujos, le pongo pintura en sus manos y él pone sus huellas en la tela. las pinturas y las temperas es lo que más le llamo la atención.

¿Ha notado algún cambio en usted o su hijo después de hacer las actividades?

Si, cambio mucho. Antes veía muchas pantallas. Lo dejábamos en la casa con sus hermanos y le ponían la tele y si no en el teléfono con los jueguitos. Y ahora no, ahora agarran la bolsita y se ponen a pintar y los hermanitos grandes también se ponen a pintar con él, ellos mismos le hacen figuritas y lo pintan. Nos ayudó bastante, lo único que no fue la pelotita. También le llenamos las bolsitas con agua y la hicimos congelar, y el la metía y la sacaba y la metía al agua y de ahí la tiro y se rompió el hielo y lo vio al cocodrilo y él lo veía. Y así él no quería pronunciar palabras, pero ahora como que se animó más.

¿Usted cambio en algo?

El prefería estar en el teléfono que hacer eso y ahora igual ve un rato la tele y luego él solito va a la mesa y empieza a pintar y a jugar. Porque antes no estaba toda la tarde viendo la tele. Y ahora no está ahí jugando y ya que se cansa mete todo a la bolsita y lo pone en un lugar donde lo hemos ensenado a poner y ahí lo deja y ya luego se echa a la cama y ya mira el teléfono y ya se duerme.

¿Le cambiaria algo a las bolsas o a las actividades?

Para mi estuvieron bien, la pelotita le gustaba meterla y luego encontrarla. Me gusto creo que fue buenísimo, buenismo, no cambiaría nada.

Mamá 2

Le pondría más colores, para que él sepa diferenciar los colores, o pinceles, los libros le cuesta un montón sentarse poner atención. Pero todo lo demás me pareció muy completo, los animales me parecieron bastante bien, la pelota ya de por si le encantaba. Me pareció muy completo. Esta bastante bien.

De pintar pinto la pared y le encanta un montón, de los colores justo nos tocaron amarillo, rojo y azul y le estuve ensenando como hacer colores, pintar si casi todo el rato esta va a otra cosa un rato, pero luego vuelve otra vez, descubrió el gusto por eso.

Yo siempre participaba con él en las actividades y pasaba tiempo con él, pero descubrí más actividades por ejemplo cuando hacia la actividad con la sabanita e hicimos la tienda él decía wow y eso estuvo muy padre.

Mamá 3

¿Cómo le fue con las actividades?

Muy bien, jugaba mucho con la pelotita, los colores me rayo todo, nos pintó todo, los animalitos, empezó con los sonidos de los animales, y sobre todo lo que era ensuciarse le encanto. La hermana abrió la bolsa y se puso a jugar con él y pintaron todo y estuvieron jugando un montón. Ella también hace como que le lee el cuento, no sabe leer, pero ella ve las imágenes y conforme a lo que ve en las imágenes le va inventando la historia. El presta mucha atención a ella, ella es su referente para todo. Pero bastante bien, bastante contenta con todo.

Noto algún cambio en el?

Eh si, si si porque ahora él se expresa más, trata de hablarte un poco más, de hacerse sentir un poco más. Por ahí te dice las palabras, él no es mucho de decir mucho papa o mama, pero ahora te dice los nombres, ella le ayuda mucho, le dice los nombres de todos nosotros, es la vocecita de él, con la bolsita didácticos la verdad que re contenta porque agarro y jugaba y se ensuciaba, es más le sacamos una foto, eran los pies, las manos, todo pintado, todo a punto de entrar al baño. El frízer la regadera todo me rayo, me rayo todo, los dos, el dirige, pero ella es la cómplice, pero jugaron un montón. El tema de los libros no tanto porque agarra y los tiras todos. Entonces esos los tengo ahora en el ropero arriba para esperar el momento en el que pueda poner más atención. Por ahí un ratito lo agarra la hermana y le va pasando las páginas y le va inventando la historia. Pero muy contenta, todo lo que sea para que él se entretenga me viene bastante bien porque es muy hiperactivo.

Todo funciono, funciono todo, con los animales aprendió los sonidos, con el león le hacía roar con el cangurito saltaba….

Mamá 4

¿Como le fue con las actividades?

Muy bien, le gustaron mucho las temperas, me llama la atención que lo usa y luego que acaba lo guarda es lo que me llama la atención. Se había manchado todas las manos, pero no había manchado nada ni la mesa ni la pared ni nada, y cuando vi él se había ido al baño a lavarse las manos, y cuando subo estaba todo limpio y estaba ahí con los animales y vino con todos los animales y ahí viene la jirafa y como su cabeza es flexible decía se murió, porque se agacho. El tema del lenguaje es el que él está más atrasado y es el que más mejoro. El libro de Lila y el agua le encanto, antes de acostarse se pone su pijama y viene y siempre duerme en la almohada con su libro, y él hace como que lo lee a su manera, le cuesta armar oraciones, pero si empezó a decir varias palabras, no forma todavía oraciones.

La telita también para escondernos y taparnos, buenismo, me encanto.

¿El cambió?

Muchísimo cambio en términos de lenguaje, no hablaba solo se tiraba en el piso y lloraba, eso también cambio mucho, es más obediente y más tranquilo, él quería agarrar sus juguetes, y ahora ya no lo quita. Estira la mano, habla mas

¿Cambio en la mama?

Los libritos, saber cómo jugar con él, como pasa el tiempo es el algo que nos ayudó mucho.

Mamá 4

¿Como le fue con las actividades?

Me fue muy bien. Mi hijo lo primero que agarro fueron los animalitos y con esos se entretuvo un montón. Y tengo una hija también de 3 años, y a ella si le gusta jugar, pero como que a él nunca le intereso jugar. Y en los libros había dibujos de animalitos y con eso empezó a preguntar y agarraba los animalitos y ahí se ponía a jugar y decía ojo, nariz, hablaba más. La pelotita no le interesó mucho. Pero los colores y todo eso si le encanto pintaba a los animales y los decoraba, y también empezó a contar lo animalitos, uno, dos, tres, no sabe contar, pero repite. Esta también aprendiendo de subir y bajar, no dice bien, pero dice subí subí. Antes no nos hablaba mucho, pero ahora si empezó a hablar más, se soltó, le empezamos a entender más.

Cambios en él?

El lenguaje mejoro mucho

¿Cambios en usted?

Yo estoy constantemente trabajando y a veces no se mucho de ellos y el viene y me dice mama, uno dos tres, o los colores, y me llama la atención, esa parte me pego mucho a tratar de ponerle mas atención a el y pasar más tiempo.

Mamá 5

¿Como le fue con las actividades?

Nos molo un montón, porque a él le cuesta hablar, entiende todo, pero no habla. Y a él le encanta la pelota, cuando la vio la pelotito, eso fue con lo que empezó a jugar primero, le ayudo a tener más comunicación con sus hermanos, tiene un hermano de 17 y otro de 13 entonces faltaba mucho que jugaran con él. Y apenas vieron todo lo que había y bueno empezaron a jugar juegos, chocho y luego vieron la pelotita y pusieron un círculo, un triángulo y él quería tirar todo adentro y saltaba, y luego le pusieron una línea en el piso y él quería caminar por ahí y ahí aprendió mucho los colores, y como le encanta la pelota quiere que le digamos los colores. Y también las tizas le gustaron mucho hace rayas. El libro le gustó, pero no tanto, un ratito y luego ya.

Pero a mí me ayudo un montón porque los otros hermanos, ya viste más grandes, ya con novia que va y que viene, pero ahora se dedican a hacer más, pasan más tiempo juntos, que les lleva la pelotita y ya saben que quiere jugar, el pizarrón que quiere pintar, los fribrones, el cuaderno, que quiere dibujar.

Cambios en él?

Si, el ahora como que quiere hablar más, hace más caso, me ayudo un montos y que se comunique más con mis hijos, le ayudo a estar más concentrado, a estarse quieto un ratito, a estar más tranquilo y lo ayuda a que haga caso.

¿Cambios en los papas?

Si la verdad porque nosotros después de 10 años, era empezar todo de nuevo, y luego yo era la única que lo hacía jugar y me ayuda a que el papa y los hermanos se involucraran y pasaran tiempo con él. Les dio idea de que hacer, ellos mismos los hermanos estaban muy metidos con los videojuegos. Y ellos mismos cuando vieron los juegos y las cosas hasta el mismo se dieron cuenta de que hay más cosas que los video juegos, e interactúan en los juegos. Una experiencia muy linda, y cuando hay mucha diferencia de edad es muy difícil. Y bueno para mí fue una experiencia para todos muy linda muy muy linda para unirnos como familia.

 Papa 6

¿Como le fue con las actividades?

Yo sí podría elegir, colores primarios, lo sume a lo que tenía en casa para enseñarle los colores, y le despertó esa parte. Puse en la mesa un mantel de plástico para jugar con la pintura y los animales congelados, pero se chorreaba. Y entonces nos fuimos a la tina, y yo estaba experimentando con el hielo, tratando de hacerlo más grande, más grueso para que durara más, hasta que hice un ladrillo grande de hielo y lo puse en la tina y a ella le encanto, decía frio frio, y se entretuvo un montón hasta que no se derritió no quiso salir. Me ayudo también porque le pude lavar el pelo y como se entretuvo con el hielo no lloro ni nada.

Cambio en el?

Conmigo es difícil. Porque el tiempo que estuve si pude jugar con ella, y estar ahí, pero normalmente no puedo por el trabajo, así que solo sería de vacaciones o los fines de semana.

Estuvo muy bueno, me gustó mucho, a mi señora también le gustó mucho, y no tenemos mucho tiempo, pero las vacaciones que tuve y hacer todas esas actividades con mi hija me gusto mucho, lo disfrute mucho.

Mamá 7

Yo soy la mama de X él tiene dos años. Y lo que me gusto de la bolsa es que capaz que parece muy básico, pero en casa no teníamos eran los animalitos que era un águila, un delfín un camello y no me acuerdo, y pues en casa no teníamos y algo tan básico le ayudo un montón veía el delfín y algo sabe que va en el agua y le hacía, así como que nadaba, el águila hacia como que volaba, el camello iba en la arena. Y luego teníamos una parte de las temperas, marcadores, crayones, que a él le encanta pintar y desde chiquito todo con la izquierda así que creo que ya es zurdo. Le encanto todo lo que es pintar. Lo de leer no tanto, como no se puede quedar quieto, el necesita moverse de acá para allá, pero no le da. La bolsa me pareció bastante completa, y tiene muchas actividades capaces que algunas parecen muy simples, pero uno no las piensa. Me dio opciones de como jugar con esto y saber que actividades simples le ayudan a mi hijo.

Papa 8

A mí me paso que uno les lee los cuentos como está escrito y de corrido y tratan de que entiendan eso y la guía que nos dieron decía que podíamos inventar ver los personajes e inventar e ir por otro camino, y eso parece que a mi hija le ayudo bastante, ella tiene problemas de lenguaje, ella no hace palabras armadas a lo sumo pone dos palabras une y ya. Y después de estar creando e imaginando esas historias empezó a poner más palabras juntas hasta me dijo pásame el payaso, tres palabras juntos, creo que todo esto de crear e imaginar la incentivo. En la bolsa nos dieron juguetes para congelar y se me ocurrió llevarla a la bañera y le tiraba agua caliente para que se desarmara y le encanto. Las temperas se las acabo en tres días, le encantaron. Pero está muy bueno como estimulación, termino todo manchado, pero fue muy estimulante para él. Yo no interactuó mucho con él porque esta acá en el día acá y yo trabajo en la tarde. Pero fue muy lindo pasar tiempo juntos y también hacer participar a la niña grande que tengo de 13 años que normalmente está en el teléfono, y se ponía a jugar con ellos. Los marcadores y los crayones muy buenos. Me pareció muy interesante, lo disfrutamos mucho.

Directora

Te diste cuenta que todos con las paredes, pero esta bueno, ayuda, están avanzando. La estimulación es muy importante. Nosotros ponemos acá las 8 horas que estamos acá pero el acompañamiento de los papas es fundamental. Que ellos vean que ustedes también un ratito están con ellos y ese ratito están haciendo algo que ustedes también los apapachan allá como nosotros los apapachamos acá. La actividad del frio les ha llamado un monto la atención a los papas. Lo primordial es que ellos sepan que pueden hacer muchas cosas, y las bolsas, más el acompañamiento que les damos nosotros acá más el acompañamiento de los papas, ¿sabes que sacamos? una generación de pum para arriba. Hubo muchos cambios, muchos cambios, chiquitos que no hablaban nada y que ahora hablan, y los papas vieron la diferencia y eso les gusta y hace que se enganchen más.

***C02***

Mamá1

Yo lo veo con mi hijo que tiene 1 ano y 5 meses y probamos primero con los libros y el esta re adaptado a los libros, él ya sabe que es un libro, que es una página, señala los colores o las figuras que hay. Entonces los libritos están muy buenos, la verdad que como son más duritos permiten que pueda sentir. Después quise probar que el dibujara en la hoja con el fibron, pero a él le llamaba más la atención del fibron era que podía insertar la tapita, con eso se quedó. Después quisimos probar lo de las cintas, pero como que fueron muy finitas entonces se perdían en su mano, quisimos probar lo del hielo, pero ya no me dio tiempo.

Yo creo que las actividades le despertaron más curiosidad en las cosas. Ahora va señala los colores, ve que hay otros diferentes creo que le sirvió un montón,

Yo aprendí a estar más atenta a los detalles, que las cosas no se dan por si solas. No es que vos le tiras un juguete y juga con eso, sino que el juguete tiene una función, por algo, un significado, que esto es un animal, es un bloque y como eso también y que yo pueda interactuar con el también, yo en mi infancia no lo tuve, en mi crianza era de crece y crece. Pero yo interactuando con mi hijo era más de enseñarle a hablar, interactuar con él por más que él no me responda como yo le estoy hablando, pero él lo está entendiendo. Las actividades fueron una herramienta, a mí no se me había ocurrido como esas cosas para que el pueda ver esos detalles y aprender, me sirvió un montón. Yo en mi casa no tengo muchas cosas, pero me sirvió un montón a mí.

Mamá 2

Para mi le sirvió mucho la herramienta. En caso de mi bebe como es chiquita primero se metía todo a la boca, pero luego ya le dije a la boca no, y empezó con los libros y las cintas, nosotros antes no leíamos libros con ella, pero creo que a mi bebe si le sirvió mucho. Aprendió con la forma de los libritos y los animalitos, miraba, observaba... aprendió muchas cosas nuevas.

A mí me sirvió en poder leer de que se trataba y ver como le ayudaban a mi bebe esas actividades. Igual, igual les agradezco mucho por esas herramientas, ¿lo tenemos que regresar?

NGG-No, no eso es para ustedes, para que jueguen con sus hijos.

Ah muchas gracias, les agradezco de verdad mucho, nos sirvió mucho. Muy buen instructivo, orienta las actividades y permite que sepamos la razón de las actividades.

Mamá 3

En el caso de mi niña, le gustó mucho jugar con la tela, a ella le encanta que yo le tape, también le gusta que yo me tape. Y tengo una hija que tiene 4 años, ella le gusta también jugar, y ya sabe que es la casa de [NOMBRE del bebé] y anda buscándola. Y también le gusto jugar mucho con la pelotita y como ya gatea entonces la persigue.

Cambio bebe

Quiere que le haga más y nos hizo jugar más, más en la noche. Y con mi hija grande también. Ya saben que en la noche es jugar con la caja, le encontramos un tiempo porque si no ellas se aburren.

A mí me sirvió para hacer más juego, más diversión, yo no sabía, esta todo en el librito y pos saber qué hacer y para que le sirve al bebe. Nos unió mucho como familia después de cenar abrimos la caja misteriosa.

Mamá 4

 A el ahora como quiere empezar a gatear, con la pelotita le iba poniendo y él se iba moviendo no gatea bien del todo, pero él iba queriendo agarrar la pelota, Después con la sabanita yo le tapaba la carita y le decía y también jugaba con el hermano de 7 años y wow. Lo demás no se lo puse porque esta muy chiquito. Pero la caja le sirvió mucho a mi bebe y compartir en familia y estar un rato juntos y si nos sirvió mucho para estar juntos.

Mamá 5

También el libro les sirve mucho, el mío tiene un año y 9 meses y él lo agarra, lo hojea, hace como si lo está leyendo y está ahí con el hermanito, y le encanta leer, ve los dibujos le digo que es  y va cambiando de hoja, mirando y le encanta. Descubrió los libros ahora con la caja, con eso, porque no le daba yo, no sabía que le gustaba, pero le encanta leer, le doy la caja y juega con eso. Le encanto la pintura y dibujar también, a veces se llena de pintura, y le encanta, pinta en casa y le encanta.

Cambio en el

Ahora se interesa más en los libros. Le encantan los libros.

Si si cuando tengo tempo ahora puedo ponerme a jugar con ellos y pasar tiempo con ellos. Nos ponemos a jugar juntos, eso no lo hacía antes.

Mamá 6

Le dimos las fibras, nos pintó a todos, pinto la casa, por todos lados, pero nos dimos cuenta que le gusta mucho jugar con las fibras, con las tizas en el piso. Leer le mostrábamos los libros, pero él no quería, él quería pasar rápido las páginas y se levantaba. Los animales sabían cuál era el cocodrilo, cuál era la jirafa, poníamos las cintas en el piso para que caminaran le mostrábamos y al principio como que no quería, como que el quería jugar a su manera, pero después ya pedía la cinta y los ponía. Eso lo empezamos a hacer cuando nos dieron la caja después del jardín, que mi marido termina de trabajar como a las 6, después de la merienda un rato nos sentamos con él en el piso a jugar y fuimos incorporando todos los días eso hasta que llego un momento en donde capaz que un día se nos olvida y estaba la bolsa colgando y el iba y nos mostraba la bolsa para jugar, pero un momento del día él quiere la bolsa, la sacamos y nos ponemos a jugar todos. Y eso nunca lo hacíamos con él. Si bien estábamos todo el día con él, no compartíamos ese juego en familia, o estaba un rato mi marido o estaba yo, o le dejábamos los juguetes y él jugaba solito o con mis nenas mas grandes. Pero esto nos involucró a estar más como familia a jugar y a enseñarle a él cosas que iba aprendiendo durante los juegos. La verdad que vimos un avance bastante grande en el que fue aprendiendo en manejar las cosas, las fibras, cada vez iba manejando mejor. A nosotros nos re sirvió la bolsa.

A nosotros nos ayudó a estar más juntos como familia en la hora del juego, en enseñarle a él cosas que aparte le enseñábamos, pero no nos dábamos cuenta eh lo que era estar un rato con el compartiendo ese momento. Y ahora con esto nos damos cuenta de que es importante porque si bien le ensenamos no es lo mismo que estar con él en ese momento y que el aprenda. Es el avance que nosotros vimos en él y en nosotros. Yo antes le daba los juguetes y me ponía a cocinar o hacer una cosa u otra. Con mis otras nenas lo pase que no jugaba con ella, no me sentaba ni un momento con ellas a jugar. Antes llegaba de trabajar, y hacia las cosas de la casa, o íbamos a la plata y les daba los juguetes y me quedaba luego con mi marido tomando mate y era como así de vayan para allá, y con esto dije con el no, con el no va a ser lo mismo. No sabíamos que era importante jugar con ellos, y a parte mi nene ya ve la bolsa y la tira, él quiere jugar con nosotros.

Mamá 7

Mi nena igual, quiere jugar con nosotros ahora, luego esta haciendo berrinche y le enseno la bolsa y se las doy y luego luego se pone tranquilo, le gusta mucho ponerse a jugar con nosotros.

Mamá 8

A mi bien, me ayudo mucho, me di cuenta lo importante que es estar con los hijos por lo menos un ratito, Porque yo casi mucho no comparto con ellos por los quehaceres de la casa y eso, pero me di cuenta de que es muy importante. Mi nena de 8 meses le gustó mucho la sabanita y sus hermanos también se involucraron y le leían y jugaban con ella y estaba lindo. Y me dieron muchas ganas de llorar, porque es cosa que yo nunca hice con mis otros hijos y está muy lindo, me ayudó mucho y me sirvió mucho para darme cuenta de lo importante que es estar y compartir con los hijos y también influyo en los hermanos para jugar juntos. Esas cosas nunca las hacía antes con mis otros hijos, yo solo sacaba los juguetes y los ponía ahí para que ellos jugaran, y ahora eso me ayudo para unirnos más. En la noche como ya están comidos entonces en ese es el momento en el que jugamos más, ahí antes de dormir jugamos un ratito con los animalitos y la mantita y se empiezan a reír y les gusta y esta rete lindo.  Mi hija está en el momento de querer gatear y con la pelotita la sigue, los animalitos también luego los usaba como cochecito. Y yo vi que ella cambio mucho, mis otros hijos también y yo también, todos cambiamos. Ahora estamos mucho más unidos.

La caja me ayudó mucho para darme cuenta de lo importante que es compartir en familia y lo bonito que es estar unidos.

Padre 9

La caja fue una experiencia muy linda donde aprendimos mucho como familia con los juegos, en pasar mas tiempo con mis hijos y compartir momentos de aprendizaje.

Mamá 10

Yo te quería comentar que yo estudio trabajo social. Entonces como que pensando desde esa área y entendiendo el contexto de la primera infancia, de las mamas y los niños que vienen acá y para mí fue re como justo lo que yo pensaba y mi maternidad fue deseada con propósito todo, pero muchas mamas no tienen eso, y la herramienta esta fue justo lo que yo pensé que estaría bueno que pudiera haber como una guía, che tienes que darle esto a tu hijo, pero si la mama no sabe cómo hacer esto, no le das como un instructivo, como lo pueden hacer, entonces esto fue la guía que yo necesitaba pero también que muchas mamas necesitaban y que son mamas solteras, que trabajan, que quieren darle algo a sus hijos pero que no pueden, porque no saben cómo, yo creo que esto les re sirvió un montón, y yo lo veo en las otras chicas. Porque ustedes no solo dieron una caja con cintas y marcadores, ustedes dieron el propósito de eso, esto funciona para esto entiendes, esto se puede hacer así, tú lo puedes adaptar, esto te ayuda un montón porque aprendes un montón, por ese lado está muy bien pensado, pensado en las problemáticas, esta muy bueno, esa es la idea, es muy importante, muy bueno. Aprendemos mucho y creo que también podemos romper malas crianzas de antes, y vamos rompiendo la generación esa, yo no tuve esto, a mí me criaron de vete a la plaza a jugar y ya no más, crece como la hierba. Y yo entiendo esa situación y la situación económica y todo lo que paso mi mamá, pero hoy tengo esta oportunidad de cambiarle esa realidad a mi hijo y como que esto me ayuda a cambiarla, me sostiene, me hace sentir que no estoy sola.

***C03***

Papá 1

A mi hija le gustó mucho la pelotita de colores, lo otro lo revolvió todo, le hice una casita con la sabanita y también le gusto, los libritos también, veía los dibujitos, pero la pelotita fue lo que más jugo. Pero jugaba un ratito y luego ya lo dejaba, es medio disperso, mi hijo tiene un año nueve meses.

Mamá 2

La tela y dibujar en el piso le llamo mucho la atención, pero la tela. Intente hacer los círculos para que ella pusiera cosas en el medio, pero siento como que no entendió muy bien lo que tenía que hacer. Pero ella más quería dibujar con los fibrones y las tizas, eso está muy bueno.  Y los cuentitos, a ella si le gusta mucho que le lea mucho, pero le gustan más los que tienen animalitos y para cantar. Y los animalitos le llaman mucho la atención, imita mucho a los animales. Y me di cuenta de que entendía, te muestra algo especifico, por ejemplo, había uno que tenía una torta y empezaba a cantar o había otro que tenía un gato y empezaba a maullar. Yo no me había dado cuenta de que entendía esas cosas y las ligaba con la vida real.

Como que le hablo y siento que ahora entiende más, interpreta que quiere decir cada cuento, la vi mucho más despierta, como que reacciona más. Lo manual como ponerse a dibujar en un cuadernito o algo así como que no le llama.

Yo le comentaría que estaría bueno quizás agregarle más imágenes de la vida cotidiana, porque como que yo veo que ella lo asimilo así, asimilo cosas de los libros con la vida real y se comunicó a través de eso en algunas situaciones. Como que estaría bien tener más imágenes de la vida cotidiana como para poder mostrarles y hablarles de eso.

La bolsa me ayudó mucho, yo le encontré la vuelta para comunicarme con ella de manera diferente, me sentía muy frustrada, yo me angustiaba, no sabía cómo enseñarle y con esto fui encontrando unas herramientas para enseñarle. Y sabe que la bolsa es para un momento en específico, también aprendió ella a guardar ella solita las cositas cuando acaba, las guarda, la cierra y me la da a mí.

Mamá 3

A mí en particular la tela fue que se la llevaba para todos lados, dibuja así. Y con lo de las cintas las pusimos en el piso para los monitos, eso como que no, prefirió pegar la cinta por todos lados en la casa.

Mi hija ya jugaba con las temperas desde antes con las hermanas, hay cosas como que ya la venia haciendo. Leer le gusta porque le leemos desde la panza, porque nosotros la lectura siempre, porque yo siempre trato de leerle. Por ahí si l e llamo la atención lo de los animalitos y los dibujos. Eso si lo disfruto mucho.

Mamá 4

Yo por ejemplo (nombre de bebe), el tema de la casita le encanto, le encanta, lo hicimos una sola vez, y después con todo lo que encuentra ella se esconde ahí, busca que nosotros le busquemos y la hermanita y nosotros ahí jugando, Donde esta <nombre.? Le digo yo y ella se ríe y se ríe, le encanta eso. Sobre el tema de la hoja, hace garabatos, no sabe qué, pero hace cualquier cosa. Y la cinta medio que no, le pone nerviosa porque también le digo yo que acá, vamos a tratar de o salirnos de la cinta y le digo no, no te vayas de lado. Y la tempera por todos lados, los juguetitos también, los libritos también le encantan, le gusta que le lean mucho. Pero si empiezas no paras por eso trato de leerle cuando está a punto de que se duerma, tiene que estar muy cansada ya porque si no no me deja, tengo que seguir, dale dale lea. Y le gusta ver los dibujitos, le doy vuelta a la hoja y me vuele de vuelta porque tiene que ver esa parte. Así que a mí me encanto, eso me re-ayudo con ella, porque así se entretiene y no anda tocando cosas que no debe tocar, por ejemplo, y jugamos a las escondidas. Y por ejemplo si mama tiene que hacer otra cosa, por ejemplo, estoy cocinando en la cocina ella, le doy la sabanita y ella se esconde en la sabanita y yo puedo estar cocinando y mientras también le busco y la tengo ahí conmigo de reojo. En ella a bolsa le despertó el interés en los libros, ahora ve un libro y quiere que le lea, antes de eso nunca le habíamos leído. Pero quiere que le leas, léele, léele, léele y no quiere que pares. Te quiere escuchar, es todo un tema con ella, y roca, cuando vos te callas empieza a roncar como de dale, dale, dale… se queja si paras. Cuando agarre el libro la primera vez, como que no le gusto, pero luego empecé a cantarle el libro y con los dibujitos, tomo ritmo y le super encanto, para mí fue una gran ayuda, porque me di cuenta de que es como que le estoy ensenando a través de eso. Porque es verdad que cuando tienes un bebe no sabes cómo ensenarle, ni que decirle, no es como que estes hola hola como estas todo el tiempo, y pues así con el libro, le lees, ve los dibujitos, te pone atención, y me doy cuenta que me pone atención, es re viva, esto fue una muy buena muy buena ayuda, muchas gracias.

Mamá5

La mía tiene 11 meses y vi que como el papel todo se lo llevaba a la boca, y entonces yo decidí cortar el papel y le hice unas bolitas. Y como no le gusta estar boca abajo yo le puse una bolita por aquí y otra bolita por acá y ya no lloraba, había como que se estiraba para alcanzar el papelito y ya que lo alcanzaba y me miraba y yo le decía muy bien, ella no gatea, mi hijo el mayo tampoco gateo directamente camino, pero yo trato de que ella utilice más sus manitas y sus piernitas entonces y después intentamos con el papel y la pintura, pero ella se lo quería meter a la boca. Entonces mi esposo me dijo por qué no intentamos con el papel, pero con la frutilla, y entonces mi esposo mismo agarraba y le ponía la manita y le decía mira la mano de (nombre de bebe) ah entonces ya. Así también probamos con la naranja y la frutilla. Eso es en cuanto al papel. Y en cuanto los cuentos, ella se metía los libros a la boca, no mi niña este es un libro mira vamos a contar, había un pajarito, y le muestro el pajarito, mira estaba volando así, y yo misma le movía las manitas para que volara así. Y mi hijo también le hacía así hermanita, mira así así, y me decía que más mami. Y yo le decía y le aumentaba había un lobo… yo no le leía el cuento yo le inventaba, y ellos decían wooow. Y ahí empezó a decir mami mami, antes solo decía agua, ahora habla mucho más. Y después en cuanto a los juguetitos, pegamos la cinta de papel, y le decía mira vamos a hacer el desfile y vamos a ver cómo, yo le hacía como que era grande yo no la veo como chiquita, y pusimos la jirafa y todos los animales, y yo no le hacia el sonido, pero le ponía en el teléfono, y le decía viste como hace? Y se reía, cada vez que el animalito hacia un ruidito se reía. Y después los sacaba y ella se los metía a la boca y cada vez que se los metía a la boca yo les ponía el sonidito. Entonces le llamaba la atención. Después tenía la pelotita que tenía el sonido y giraba y giraba, y ella miraba para un lado o para el otro lado. Y todo ella lo hace bailando, porque siempre tenemos música de fondo la música de mi esposo que es de la India, y así baila ella, mi niño y yo también les sigo. Después esto no estaba en el libro o las hojas, pero puse una botella con agua le puse un clip con forma de pescadito y cuando apretaba el pescadito subía. Y después le puse un lapicero y dos tapitas y le jalaba con una cinta y hacia como que el carrito corría. Y se reía a carcajadas.   En cuanto a la tela, jugábamos a escondernos con ella, y luego ya no usábamos la tela nos escondíamos en otro lado y ella nos buscaba y el hermanito jugaba también con ella. Pero muy interesante los libros, todo muy didáctico, y como que despierta más la… antes no estaba muy estable para sentarse, pero ahora ella misma quiere agarrar los animalitos, ella quiere alcanzar con sus manitas las cosas, y los colores le llaman la atención. Le sirvió muchísimo a ella porque ahora encima ella se sostiene y se mantiene mucho mejor.

Reseacher-Esta super bien como adaptaste y extendiste? ¿Y eso era algo que hacías antes con tu hijo?

 No eso fue algo que surgió a partir de esto, yo no hacía nada de esto con mi hijo. Y eso es algo que yo hago, siempre trato de ponerle más, de hacer algo más de lo que me piden en mi trabajo, A veces me dicen solo cámbiale la batería, pero yo le veo y si necesita algo más siempre trato de hacer más de lo que piden, Así que de la misma manera yo leía, miraba y decía ah no, también puedo hacer esto, por ejemplo agarre botellitas de esas donde viene la medicina ya viste como el ibuprofeno y todo eso, las lave y en uno metí lentejitas, en otro piedritas y los sonidos son distintos y ver que sonido le gusta más. Y con la música agarra el que le gusta y empieza a moverse y bailar.

A mí me llena de mucha felicidad que le propongo las actividades y ella quiere más, luego duerme solo 15minutos y se despierta porque ella quiere hacer otra actividad.

Directora

Dos docentes quieren compartir su experiencia con las cajas de Oxford, porque ellas se sentaron a jugar con estas cajas, y entonces les cuento, digo esto fue puntapié, esto te incita a…, vos podés volar y empezar…, esto es de acá partimos, no sé a dónde llegamos. Ella tomo un niño de su sala que ella escogió. Y nos va a contar la docente su experiencia que fue maravillosa, pero el niño solo al ver todo esto hizo un poquito más y de ahí se siguió...

Docente 1 (sala de 2 y 3)

Nosotros preguntamos en la sala quién tenia ganas de salir un ratito y hacer una actividad a parte y fueron varios que levantaron la mano y bueno lleve a todos, también unos chicos de dos, y bueno me senté a jugar los invite a ver que surgía, leí los libros, lo de la casita todo. Plantee una actividad en la mesa y la llevaron a cabo y les encanto, pero pedían más esto es lo que sucede que cuando algo los atrae piden más. Y esta bueno poder habilitar una entrada abierta a esas actividades posibilidades porque abre un montón de cosas, y hubo una en particular que hizo que era el juego de las              , y yo le iba destacando e iba descubriendo les sacaba uno les sacaba otro. Pero Después me pedían que ellos querían ordenarlo solos, y querían hacer que lo sacara, lo pusieran de una manera, lo mostrara de una manera, lo mezclara con la sabana y lo volvían a poner. Y como así se fueron dando varias actividades y pautas que ellos mismos las iban planteando, yo estaba abierta para lo que ellos me proponían, y la verdad que salió muy enriquecedor porque cuando volvimos a la sala ellos mismos les contaban a los compañeros, viste el juego… y los demás me pedían. Y yo te iba a pedir si me prestan la caja para los otros 16 que también lo quieren hacer. También lo hicimos con la sala de dos, plantee la actividad del libro, la de los animales y ellos me lo empezaron a nombrar, me hacían todos los sonidos, después me nombraban los colores, se fueron abriendo un montón de oportunidades que está muy enriquecedor y muy lindo con los chicos. Así que más que agradecida por las oportunidades que nos dan, porque también nos sirve a nosotras para abrirnos y darnos más ideas y empezar a incorporar esas actividades, esta bueno abrirse un poco más. Me ayudo a darme más ideas y a darles la oportunidad a los chicos de que sean ellos los que guíen el juego, es lo especial de eso, uno les puede plantear una actividad, pero si ellos después van lo van incrementando y se ponen más desafíos es mucho enriquecedor para ellos y para nosotros que lo estamos mirando.

Docente 2 (sala de 1)

Aquí en la sala tenemos entre 8 meses a 2 años. También les propusimos a los chicos salir a hacer las actividades. Una nena en particular le propusimos hacer el juego de las cintas de colores con los animales y la nena al principio notaba que había algo, pero no correspondía al animal. Y entonces me miraba, y empezó a nombrar a los animales y hacer los sonidos, incluso empezó a apilar uno encima del otro, entonces había otra propuesta. Y después en la sala tratamos el cuento que se va abriendo, lo hicimos en la mañana cuando están más tranquilos, y ese cuento nos llevó a una canción, y a hacer los sonidos, y si no los saben ellos los inventan, empezaron a decir tiene manos, tiene pies, ¿cuántas patas tiene? La imaginación de ellos es muy enriquecedora así que la verdad nos sirvió un montón, la vamos a seguir usando, la vamos a pedir prestada si es necesario porque es muy lindo ver la imaginación de los nenes de un juego simple que les propongamos. Los dejamos ser libres y que ellos puedan experimentar y que nos ensenen a nosotros porque nosotros estamos para aprender.   Respecto a las pinturas también, fuimos explorando las temperas, y todo empezamos con los lápices, el crayón, y así y hoy por hoy los nenes ya pintan sus carpetas ellos solitos ósea los dejamos y que ellos se expresen. Así que les agradecemos un montón y vamos a seguí usando la caja, seguir trabajando con la caja y todo porque es muy lindo verlos crecer.

Mamá 6

La verdad es que nos sirvió un montón la caja. Mi niño tiene un año tres meses, le mucho la tempera y los crayones, le gusta mucho pintar, pero en papel no en la tela. Le gusta pintar, agarra los cuadernos del hermano y empieza a pintar todo lo que hay. Los animalitos, tiene un dinosaurio que le abre la boquita y empieza a hacer ruido y le encanta, también venia un monito y un león, y le digo como hace el monito y hace oo oo oo y el león todavía no le sale, pero todos los días intenta hacer el sonido de los animales, le sirvió muchísimo, ahora está diciendo muchas más cosas, se le soltó el lenguaje.  Y después lo ahora tú le decís agarra la cosa y va y lo hace, entiende mucho más que antes. El librito también le llama mucho la atención los colores y los dibujitos. A partir de la caja le empecé a leer los cuentos, eso nunca lo hice con el hermanito grande. Igual ahora el mismo agarra el librito y me lo trae para que se lo lea, el del Ratón Gastón, lo trae y me lo muestra y yo se lo voy contando y le digo quienes son. A mí me gustó mucho compartir el tiempo leyendo con él, como que yo también voy aprendiendo voy aprendiendo de vuelta. Y la casita, le pusimos dos banquetas y arriba la tela, y él se va y se mete ahí y mete todo lo que encuentra, zapatos, todo, todo abajo. Y el hermano juega con él.  Va aprendiendo mucho, la verdad que lo disfrutamos mucho. Muchas gracias

Papá 7

Yo un poco también como que a uno se le acaban las ideas para jugar, no, y esto te da un poco más de ideas, lo de la tela de ponérsela, las temperas (inaudible), lo que no le gusta es guardar, y la verdad que sí que si me ayudo un montón a tener más ideas que hacer lo de las cintas. La verdad que muy bien

Mamá 8

A mí me ayudo bastante, yo tengo cuatro hijos y con ninguno de mis hijos tuve ninguna actividad, no me acuerdo que yo me haya sentado con mis hijos, darle atención para jugar, ayudarla y (nombre de bebe) es como que… A mí me reclaman los hermanos, que yo nunca haya jugado con ellos y ahora con ella sí. Bueno que pasa, ahora que yo recibí la caja, no es para llevar a guardar como dice la directora, así que fui y me senté con [NOMBRE del bebé] y estábamos nosotras dos solas, y llega el hermano me dice que hacen? Acá jugando con [NOMBRE del bebé] a ver qué hace ella. Y claro ahí vienen los reclamos, y es verdad porque nunca le puse la atención de jugar, de darle importancia a sus ideas, no les di atención. Y no sé por qué, porque en ese momento yo no estuve trabajando. Estuve con ellos todo el tiempo, pero no de sentarme a jugar o darles atención, darles tiempo que es lo que ellos necesitan cuando van creciendo. A mí me ayudó mucho con [NOMBRE del bebé] porque yo veo que ella está aprendiendo a hablar cuando ve los dibujitos, agarra un juguete y va te dice, y te lleva el juguetito, y que pasa cuando lleva el juguetito y quiere que este con ella ahí jugando. Pero que pasa que yo tengo un hijo de 5 años que es re celoso, cuando me pongo a jugar con [NOMBRE del bebé] me dice no me amas, cuando estas con [NOMBRE del bebé] no existo. Y no es así, lo que pasa es que ella es chiquitita ella va aprendiendo jugando, pero me dice tu no lo haces conmigo, y a mi me da mucho nervio porque yo ya no estoy todo el tiempo con ellos, hace 3 años que yo empecé a trabajar y ya no estoy todo el día con ellos, yo no estoy en la casa, la hora de conversar con ellos es la hora de la cena. Nos sentamos a cenar y a conversar, pero el padre quiere comer tranquilo y comer silenciosamente. Y le digo no, este es el momento donde nosotros podemos darles un poco d atención y saber qué es lo que pasa en la escuela, en el jardín… ese es el momento porque después debemos acostarnos y luego yo me levanto a las 4.30. Y ese es el reclamo que ellos creen que yo estoy con [NOMBRE del bebé] todo el tiempo, pero ellos no saben que yo la dejo en el jardín, me voy a trabajar le retiro y vuelvo para casa. La caja me vino muy bien, porque como le digo me dio la idea de sentarme y darles un poquito además de cariño, darles más importancia, de importarme me importan todos mis hijos, pero me empuja a hacer un poco de actividad con ellos y pasar más tiempo con ellos. La caja es muy buena idea, que buena idea tuvieron de regalarle la caja, me dio muchas ideas, que podemos compartir y las que no sirvieron nos sirvieron para hacer otras cosas para darnos más ideas, muy bueno. Me dejo pasar tiempo de calidad con mi hija. Yo me pregunto por qué no hice esto antes con mis hijos, no trabaje, tuve mucho más tiempo para hacer esto, fue mi error, lo reconozco, no sabia, pero me duele que ahora me digan por qué no hiciste eso conmigo, pero ahora trato de hacer algo con cada uno de ellos, de pasar tiempo, darle atención y estar ahí para ellos.

Mamá 9

La caja nos ayudó mucho, yo tuve un accidente, perdí la memoria, perdí muchas cosas y a él me aferré mucho y lo sobreprotegía y yo lo limitaba, lo limitaba y lo limitaba… no quería que le pasara nada. Yo estaba muy dudosa muy dudosa de hacer cosas, no me sentía segura, después no dieron la caja y ahí si ahora se levantaba a las 6 de la manzana, mami, mami ya es hora y se levantaba para jugar. Y ahora al darnos la caja yo cambie mis expectativas, y también cambio su papa, él se sentaba, la cargaba y como que no sabía qué hacer con ella, pero ahora él se pone a hacer las actividades, el mismo saca la caja, saca a los animales y pasa realmente tiempo de calidad con ella, aunque sean 5 o 10 minutos, pero se lo está dando a la nena. pero pues ese también es un alivio para nosotras, porque me da un poco de tiempo de hacer otras cosas, y ya entonces yo creo que es super super super, para que ellos también sean participes de la crianza de los hijos, pero a veces la mujer carga con todo porque ellos no se involucran. Y yo también incluyo a su hermanito más grande y él tiene otras ideas y lo incluimos, lo trato de que él también sea parte de esto. Y mi niña también se siente muy feliz. La verdad que muy muy interesante la caja, me dio muchas ideas, muchas ideas para poder hacer, para ayudarle a crecer, me ayudo a sentirme más segura, la verdad que una gran ayuda para mí como mamá.

Papá 10

Yo por ejemplo, la caja también me ayudó mucho para organizar toda la actividad en un recipiente. Nosotros ya teníamos muchos juguetes para él, lo tenemos todo a la mano así que él va y saca. En nuestra caja nos tocaron los animales marinos así que lo incluimos a la hora del baño, por ejemplo, la ballena tenía una buena capacidad así que al ser más grande se alimentaba de otros peces, jugaba con la foca… le gustó mucho. Participaba, jugo mucho con el tema de la tela, utilizaba el tiempo libre para dibujar, le gustaba más los lugares abiertos así que iba al balcón y le encantaba ensuciarse, y pues es una buena etapa para que disfruten de eso. También le ensenamos que todas esas actividades cabían en la caja así que él tenía que buscarle la manera para que todo cupiera de vuelta ahí en la caja, muy organizado el dejaba todo ordenado al final. A él le gusta mucho hacer cosas nuevas, como todo chico, así que después de un rato propone hacer otras actividades con esos mismos juguetes. También incluimos a los otros dos hermanos, siempre participaban con él, le ayudaban con los ruidos. En cuanto al libro le pareció bastante interesante ver las diferentes imágenes, yo creo que le ayudo a estar más atento y más coordinado con lo que quiere hacer. Vimos la propuesta bastante positiva. Nosotros cotidianamente ya les dábamos su tiempo a nuestros hijos, por ejemplo, los grandes juegan al cludo, él no se sabe las reglas, pero quiere de todas formas participar. Pero nos gustó mucho la propuesta, nos dio nuevas ideas.
