# Supplementary material for: Shaping Development Through Parents: A Scalable Approach to Early Learning
Source: Dev Sci. 2026 Sep 4;29(6):e70279. doi: 10.1111/desc.70279 (PMC13543324; doi:10.1111/desc.70279)
Supplement: Supplementary file 5 [file DESC-29-e70279-s005.docx]

**Supplementary Material X**

Cross-context Adaptation of Playful Packs: UK vs Argentina

| **Component** | **Worked well in UK (retained in Argentina)** | **Required adaptation in Argentina** | **Not feasible / removed in Argentina** | **Adaptations introduced in Argentina** |
| --- | --- | --- | --- | --- |
| **Overall approach** | Play-based, low-cost, easy-to-implement activities; focus on enjoyment rather than outcomes | Maintained, but adjusted for slightly younger age group and local caregiving practices | — | Greater emphasis on flexibility for infants (4–36 months) |
| **Materials** | Use of simple, tangible materials (e.g., paints, toy animals) to facilitate play | Adapted due to cost and availability constraints | Some materials difficult to source locally at low cost | Packs assembled with locally appropriate materials where possible |
| **Activity design** | Open-ended, adaptable activities that allow child-led play | Instructions simplified and adapted linguistically and culturally | Some activities less relevant to local context | Added clearer suggestions for adapting activities to child age and interests |
| **Language and format** | Simple, easy-read activity cards | Translated into Rioplatense Spanish and culturally adapted | — | Adjusted wording and clarity to match local language use and literacy levels |
| **Age range** | Designed for children aged 8–36 months | Expanded to include younger infants (4–36 months) | Some activities less suitable for very young infants | Added new activity (peekaboo with fabric) for younger babies |
| **Outdoor activities** | Activities such as bug hunts feasible and appropriate | — | Outdoor exploration activities excluded due to environmental safety concerns (e.g., contamination risks) | Greater focus on indoor activities |
| **Books and literacy activities** | Encouraged shared reading and early literacy | Maintained, but required adaptation for attention span and developmental readiness | Some parents found books difficult to use with very young or active children | Encouraged flexible use (e.g., storytelling, adapting book use) |
| **Implementation context** | Designed for relatively stable home environments | Adapted to contexts with greater socioeconomic constraints | — |  |
