# Supplementary material for: Shaping Development Through Parents: A Scalable Approach to Early Learning
Source: Dev Sci. 2026 Sep 4;29(6):e70279. doi: 10.1111/desc.70279 (PMC13543324; doi:10.1111/desc.70279)
Supplement: Supplementary file 6 [file DESC-29-e70279-s006.docx]

**Supplementary Material 6**

**Transcript of Parent Feedback Sessions (English translation)**

***1st CPI (Centro de Primera Infancia)  Early Childhood Center 1***

**Mom 1**
Because he decorated my wall, because everyone says to him “Oh chubby, what did you do? You made a Picasso.” He drew a big circle with little lines like this.

**What activities did he like the most?**
The painting, the paints. He learned to write his name. I draw for him, put paint on his hands, and he stamps his handprints on the fabric. The painting and paints are what caught his attention the most.

**Have you noticed any changes in yourself or your child after doing the activities?**
Yes, a lot changed. Before, he spent a lot of time in front of screens. We used to leave him home with his siblings and they’d put on the TV, or he’d be on the phone playing games. But not anymore. Now they grab the activity bag and start painting, and even his older siblings join in. They make little shapes for him and paint together. It helped a lot. The only thing he didn’t like much was the little ball. We also filled the bags with water and froze them, and he would put them in and take them out of the water, and then he threw it and the ice broke and he saw the crocodile inside. That really captivated him. Before, he didn’t want to speak, but now he’s more encouraged to.

**Did you change in any way?**
He used to prefer the phone over this, but now he still watches a little TV and then goes to the table by himself to paint and play. Before, he’d spend all afternoon in front of the TV. Now, once he’s tired of playing, he puts everything back in the bag and places it where we taught him to keep it. Then he lies down, watches the phone, and goes to sleep.

**Would you change anything about the bags or activities?**
For me, they were great. He liked putting the little ball in and then finding it. I really liked it, I think it was amazing. I wouldn’t change anything.

**Mom 2**
I would add more colors so he can learn to differentiate them, or maybe paintbrushes. He has a hard time sitting and paying attention to books. But everything else seemed very complete. The animals were really good. He already loved the ball. It was all quite good.

He painted the wall, and he loved it. We happened to get yellow, red, and blue, so I was teaching him how to mix colors. He paints almost all the time, he takes breaks and then comes back. He discovered he really enjoys it.

I always participated with him in the activities and spent time with him, but I discovered more things to do. For example, when we did the blanket activity and made a little tent, he said “wow,” and that was really special.

**Mom 3**
**How did the activities go?**
Very well. He played a lot with the little ball, and the colors—he scribbled on everything, painted everything. He started doing animal sounds, and he especially loved anything messy. His sister opened the bag and played with him. They painted everything and had a great time. She even pretends to read him the story—she doesn’t know how to read, but she looks at the pictures and makes up a story. He pays close attention to her; she’s his role model. I’m really happy with everything.

**Did you notice any changes in him?**
Yes, definitely. Now he tries to express himself more and communicate more. He used to hardly say "mom" or "dad," but now he says names. She helps him a lot, tells him everyone’s names. She’s like his little voice. The activity bag was great—he would grab it, play, get all messy. We even took a photo—his feet and hands were all painted, about to go into the bath. He drew all over the freezer and the shower, everything. He’s the ringleader, but she’s his accomplice. They played so much.

Books, not so much—he tends to throw them. So now I keep them in the closet until he’s ready to pay more attention. Occasionally his sister grabs one, turns the pages, and makes up stories for him. But I’m very happy—anything that entertains him is great because he’s very hyperactive.

Everything worked. He learned animal sounds—he’d roar like a lion, hop like a kangaroo...

**Mom 4**
**How did the activities go?**
Really well. He loved the paints. What surprised me was that he’d use them and then put them away. He got paint all over his hands but didn’t dirty the table, the wall, or anything. I saw he had gone to the bathroom to wash his hands, and when I went up, everything was clean. He was playing with the animals and brought them to me—“Here comes the giraffe”—and since its head is flexible, he said “it died” because it bent down.

Language is where he’s the most delayed, and that’s where he improved the most. He loved the book *Lila and the Water*. Before bed, he puts on his pajamas and always goes to bed with the book, pretending to read it. He struggles to make full sentences, but he started saying several words—still not full sentences yet.

The little blanket we used to hide and cover ourselves? Wonderful. I loved it.

**Did he change?**
A huge change in language. He didn’t talk, just threw himself on the floor and cried. That’s changed a lot. He’s more obedient and calmer. He used to snatch toys, now he doesn’t. He reaches out and talks more.

**Did you change?**
The little books, learning how to play with him, how to spend time—that helped us a lot.

**Mom 5**
**How did the activities go?**
We loved it. He struggles to speak—he understands everything, but doesn’t talk. He loves balls, and when he saw the little ball, that’s what he played with first. It helped him communicate more with his siblings—one is 17 and the other 13—and they didn’t really play with him before. Once they saw everything in the bag, they started playing games. He was thrilled. They made a circle and triangle, and he wanted to throw the ball into them and jump. They drew a line on the floor and he wanted to walk on it. He learned colors that way, and since he loves the ball, he asks us to name the colors. He also really liked the chalk—he draws lines with it.

The book—he liked it, but just for a little while.

But it helped me a lot, because the older siblings—teenagers with girlfriends, coming and going—they now spend more time with him. They know he wants to play when he brings the ball, or the chalkboard, or the crayons.

**Changes in him?**
Yes, now he wants to talk more, listens better. It really helped his communication with his brothers. He’s more focused, sits still longer, and is calmer and more obedient.

**Changes in the parents?**
Yes, for sure. After 10 years, it felt like starting over. I was the only one playing with him, but this helped get his dad and siblings involved and spending time with him. It gave them ideas. The older boys were really into video games, but now they saw there’s more than just screens. It was a beautiful experience for all of us, especially with such a big age gap—it brought us together as a family.

**Dad 6**
**How did the activities go?**
If I could choose, the primary colors—I added what we had at home to teach her colors. I put a plastic tablecloth on the table to play with the paints and frozen animals, but it got messy. So we went to the tub, and I started experimenting with making thicker ice so it would last longer. Eventually, I made a big ice block and put it in the tub. She loved it, kept saying “cold, cold,” and wouldn’t get out until it melted. It also helped because I could wash her hair while she played and she didn’t cry or fuss.

**Change in her?**
With me, it’s harder—because when I was there, we did get to play, but usually I can’t because of work. So only during vacations or weekends.

It was great. I really liked it, my wife did too. We don’t have much time, but during the vacation, doing those activities with my daughter was really nice—I enjoyed it a lot.

**Mom 7**
I’m the mom of X, he’s two. What I liked about the bag is that even though it seems basic, we didn’t have those little animals at home—a dolphin, eagle, camel, I forget the others. Just something that basic helped him a lot. He saw the dolphin and somehow knew it went in water, and would pretend to swim. The eagle—he made it fly. The camel walked in the sand.

Then we had paints, markers, crayons—he loves to paint. Since he was little, he’s always used his left hand, so I think he’s a lefty. He loved everything to do with painting.

Reading—not so much. He can’t sit still, he needs to move around.

I thought the bag was very complete. Some activities may seem simple, but we don’t think of them. It gave me new ways to play and simple activities that help my child.

**Dad 8**
What happened to me was that I used to read stories just as they’re written, straight through, and tried to get her to understand that. But the guide we got said we could invent—look at the characters, make things up, take a different path. That really helped my daughter. She has language delays—she doesn’t make full words, maybe just two-word phrases. But after creating and imagining those stories, she started putting more words together. She even said, “pass me the clown”—three words. I think all that creative play really encouraged her.

In the bag, we got toys to freeze, and I took her to the bathtub and poured hot water on the ice so it would melt. She loved it. She used up all the paints in three days—loved them.

It was great stimulation—she ended up covered in paint, but it was very enriching for her.

I don’t usually interact much with her because she’s here during the day and I work in the evening. But spending time together was really nice, and getting my older daughter (13) involved was great too. Usually she’s on her phone, but she played with them. The markers and crayons were excellent. We really enjoyed it.

**Director**
Did you notice how they all painted the walls? But that’s great—it helps, they’re progressing. Stimulation is very important. We’re here 8 hours a day, but the parents’ involvement is key. The children need to see that you also spend time with them, and during that time, you’re doing something loving—just like we do here.

The cold/ice activity really caught the parents’ attention. The most important thing is for them to realize they can do so many things. The bags, plus our support, plus the parents’ involvement—what do we get? A generation that takes off. There have been many changes. Kids who didn’t speak at all now do, and the parents noticed the difference, which makes them even more engaged.

**2nd CPI (Centro de Primera Infancia)  Early Childhood Center 2**

**Mom 1**
I see it with my son who is 1 year and 5 months old. We tried starting with the books, and he adapted really well to them. He already knows what a book is, what a page is; he points to the colours or the shapes. So the little books are really great—honestly, because they’re sturdier, he can really feel them.
Then I wanted to try letting him draw on paper with the marker, but what caught his attention more was that he could put the cap on and off—that’s what kept him engaged.
Then we tried the ribbons, but they were so thin they got lost in his hand. We wanted to try the ice activity, but I didn’t have time.

I think the activities sparked more curiosity in him. Now he goes around pointing at colours, he sees that there are different ones—I think it really helped him a lot.

I learned to pay more attention to the details, that things don’t just happen on their own. It’s not like you throw a toy at them and they play with it—no, the toy has a function, a purpose, a meaning. Like this is an animal, this is a block—and I also realised that I can interact with him too.
In my own childhood, I didn’t have that. My upbringing was just about growing up and growing up.
But interacting with my son, it became more about teaching him to talk, interacting with him—even if he doesn’t respond the same way I’m speaking to him, he still understands.
The activities were a tool. I hadn’t thought of doing those kinds of things so he could notice those details and learn. It was really useful. I don’t have many things at home, but this really helped me a lot.

**Mom 2**
For me, the toolkit was really useful. In my baby’s case, since she’s very little, at first she would just put everything in her mouth. But then I told her “not in the mouth,” and she started using the books and ribbons.
Before, we didn’t read books with her, but I think the toolkit really helped her. She learned from the shape of the little books and the little animals—she looked, she observed... she learned a lot of new things.

For me, it helped to read what the activity was about and see how it helped my baby. Really, thank you so much for this toolkit. Do we have to return it?

**NGG:** No, no, that’s for you to keep, to play with your children.

Ah, thank you so much! Really, thank you—it helped us a lot. The instructions were very good, they guide the activities and help us understand the reason behind them.

**Mom 3**
In my daughter’s case, she really liked playing with the fabric. She loves when I cover her with it, and she also likes when I cover myself.
I also have a 4-year-old daughter—she likes to play too, and she already knows it’s the baby’s house [referring to the play setup] and goes looking for it. She also really liked playing with the little ball. Since the baby is crawling now, she chases after it.

**Change in the baby**
She wants me to do more and made us play more, especially at night. And with my older daughter too. Now they know that at night we play with the box—we found time for that because otherwise, they get bored.

For me, it helped to play more, have more fun. I didn’t know what to do before, but it’s all in the little book—it tells you what to do and what it’s for. It brought us closer as a family—after dinner, we open the mystery box.

**Mom 4**
Now that he wants to start crawling, I placed the ball in front of him, and he started moving—he doesn’t crawl fully yet, but he tries to get the ball.
Then with the little blanket, I’d cover his face and talk to him. He also played with his 7-year-old brother, and it was like “wow.”
I didn’t use the other activities because he’s still very little. But the box really helped my baby—and sharing time as a family, being together for a while—it really helped us spend more time together.

**Mom 5**
The book helped a lot too. My son is 1 year and 9 months old—he picks it up, flips through it, pretends he’s reading, and sits there with his little brother. He loves reading, looks at the drawings—I tell him what they are, and he turns the page, looking, and he loves it.
He discovered books now with the box—because before, I didn’t give them to him, I didn’t know he liked them. But he loves reading. I give him the box and he plays with it.
He also loved the painting and drawing—sometimes he gets covered in paint and he loves it. He paints at home and really enjoys it.

**Change in him**
Now he’s more interested in books. He loves books.

Yes, yes, when I have time, I can now play with them and spend time with them. We sit down and play together—I didn’t do that before.

**Mom 6**
We gave him the markers—he painted all of us, the house, everywhere. But we realised he really enjoys playing with the markers, and with chalk on the floor too.
We showed him books, but he didn’t want them—he just wanted to flip the pages quickly and get up.
He knew which animals were which—he could tell the crocodile from the giraffe.
We put the ribbons on the floor for him to walk on—we showed him—but at first, he wasn’t interested. He wanted to play his own way. But then he started asking for the ribbons and placing them himself.
We started doing that when we got the box—after nursery, when my husband finishes work at 6, after snack time we’d sit on the floor with him to play.
We started incorporating it every day, until one day we forgot and the bag was hanging up—he went and showed us the bag to play.
Now it’s part of his routine—he wants the bag. We take it out and all of us play together.
And we never did that before with him. Even though we were with him all day, we didn’t share that kind of play as a family. Either my husband played with him for a bit, or I did, or we just gave him toys and he played on his own or with his older sisters.
But this made us get more involved as a family, to play and to teach him things that he learned through play.
Honestly, we saw a big improvement in how he handled things, the markers—he’s getting better at using them. The bag really helped us a lot.

It helped us be more together as a family during playtime, to teach him things we were already teaching but without realising the importance of spending that moment with him.
Now with this, we realise how important it is—because even if you’re teaching him, it’s not the same as being there with him and having him learn that way.
That’s the progress we saw in him—and in ourselves. Before, I’d give him toys and go cook or do something else.
With my other girls, I didn’t play with them—I didn’t even sit with them to play. I’d come home from work, do chores, or we’d go to the park, give them toys, and then my husband and I would sit and drink mate—it was like “you kids go over there.”
But this time I said, “not with him, it won’t be the same.”
We didn’t know how important it was to play with them.
Now my little boy sees the bag and throws it down—he wants to play with us.

**Mom 7**
My little girl is the same—now she wants to play with us.
When she’s having a tantrum, I show her the bag and give it to her, and she immediately calms down.
She really likes playing with us.

**Mom 8**
It was good for me—it helped me a lot. I realised how important it is to spend at least a little time with your children.
Because I usually don’t spend much time with them, with all the housework and everything—but I realised it’s really important.
My 8-month-old daughter really liked the little blanket, and her siblings got involved too—they read to her and played with her, and it was lovely.
It made me want to cry, because these are things I never did with my other kids.
It really helped me and made me realise how important it is to be present and share time with your kids—and it also got the siblings to play together.
I never did that kind of thing before with my other kids. I just took the toys out and left them there to play.
But this helped us become more united.
At night, once they’ve eaten, that’s our time to play—just before bedtime.
We play a bit with the little animals and the blanket—they start laughing, they enjoy it, and it’s really lovely.
My daughter is at the stage where she wants to crawl, and she chases the little ball. She even used the little animals like toy cars.
And I saw a big change in her, in my other kids, and in myself—we all changed.
Now we’re much more united.
The box helped me realise how important it is to share time as a family and how beautiful it is to be close and connected.

**Dad 9**
The box was a really lovely experience. We learned a lot as a family through the games—spending more time with our children and sharing learning moments together.

**Mom 10**
I wanted to tell you—I study social work.
So from that perspective, understanding the context of early childhood and the moms and children who come here—for me, this was exactly what I had been thinking about.
My own motherhood was planned and intentional and everything, but many moms don’t have that.
And this toolkit was exactly what I had imagined would be helpful—like a guide.
“Hey, you need to give this to your child,” but if the mom doesn’t know how to do that, if you don’t give her instructions, she won’t know how.
So this was the guide I needed—and that many moms need.
There are single moms, moms who work, who want to give something to their kids but don’t know how.
I think this really helped them a lot—I see it in the other moms.
Because you didn’t just give out a box with ribbons and markers—you gave us the *purpose* behind it. “This works for this reason. You can do it this way. You can adapt it.”
It helps so much because you learn a lot.
From that angle, it’s really well thought out—thought out with the challenges in mind.
It’s great. That’s the idea—it’s very important, really good.
We learn a lot, and I think it also helps us break harmful parenting patterns from the past.
We’re breaking that cycle—I didn’t have this. I was raised like, “go to the park and that’s it,” grow like a weed.
And I understand that situation, the economic challenges and everything my mom went through—but today, I have the chance to give my child a different reality.
And this helps me do that. It supports me. It makes me feel like I’m not alone.

**3er CPI (Centro de Primera Infancia)  Early Childhood Center 3**

**Dad 1**
My daughter really liked the colourful little ball, she mixed everything else up, I made a little house with the blanket and she liked that too, and the little books too—she would look at the pictures—but the ball was what she played with the most. But she would only play for a little while and then stop, she's a bit scatterbrained. My son is one year and nine months old.

**Mum 2**
The fabric and drawing on the floor really caught her attention, especially the fabric. I tried doing the circles so she could place things in the middle, but I feel like she didn’t really understand what she was supposed to do. But she really just wanted to draw with the markers and chalk—that was really great. And the little books—she really likes being read to a lot, but she prefers the ones with animals and songs. Animals really catch her attention, she imitates them a lot. And I realised that she understands—she points to specific things. For example, one book had a cake and she would start singing, or another had a cat and she would start meowing. I hadn’t noticed before that she understood these things and connected them to real life.

When I talk to her now, I feel like she understands more—she interprets what each story is about. I noticed her much more alert, more responsive. Things like sitting down to draw in a notebook don’t really appeal to her.
I would suggest maybe adding more images of everyday life because I can see that she processes that way—she connects things from the books to real life and uses that to communicate in some situations. I think it would be good to have more images from everyday life, so we can show and talk about them.

The bag really helped me—I found a new way to communicate with her. I used to feel very frustrated, I was overwhelmed, I didn’t know how to teach her, and this gave me tools to do that. And she knows now that the bag is for a specific moment—she’s learned to tidy up by herself when she finishes. She puts the things away, closes the bag, and hands it to me.

**Mum 3**
For me, the fabric was the favourite—she carried it around everywhere, drawing like that. And with the ribbons, we put them on the floor for the little monkeys, but she wasn’t into that. She preferred sticking the ribbon all over the house.

My daughter already played with paints with her sisters before, so some of these things she was already doing. She likes reading because we’ve been reading to her since I was pregnant—reading has always been part of our routine. Maybe what caught her attention were the animals and the pictures. She really enjoyed that.

**Mum 4**
For me, (baby's name), she loved the little house—we only made it once, but ever since then she hides in it with whatever she can find. She wants us and her sister to look for her and play. I say, “Where’s (baby’s name)?” and she just laughs and laughs. She loves that.

About the paper—she scribbles, doesn’t know what she’s doing exactly, but she makes something. The tape, not so much—it made her a bit nervous. I also tell her, “Let’s try not to go outside the tape,” and she gets frustrated. She spreads the paint everywhere, and the toys and the books too—she loves those. She loves being read to a lot, but once you start, you can’t stop. That’s why I try reading when she’s about to fall asleep. She has to be very tired, otherwise she won’t let me stop: “Keep reading! Keep reading!” She loves looking at the pictures—if I turn the page, she flips it back because she wants to see that part again.

I really loved this—it helped me so much with her. It keeps her entertained and she doesn’t go around touching things she shouldn’t. We play hide and seek, and for example, if I’m doing something else—cooking in the kitchen—I give her the little blanket and she hides under it, and I can keep an eye on her while I cook.

The bag sparked her interest in books—now when she sees a book, she wants me to read it to her. We hadn’t read to her before this. Now it’s “Read to me! Read to me!” and she doesn’t want me to stop. She wants to listen. The first time she grabbed a book she didn’t like it much, but then I started singing the book to her, and with the pictures, it caught on and she absolutely loved it. For me, this was a huge help—I realised that through this, I’m actually teaching her. Because when you have a baby, you don’t know how to teach them or what to say—it’s not like you just go around saying “Hello, how are you?” all the time. But with the book, you read, she looks at the pictures, pays attention, and I can see she really does. She’s very sharp. This was a really good tool, thank you very much.

**Mum 5**
Mine is 11 months old, and I saw that she just put all the paper in her mouth. So I decided to tear it into little balls. Since she doesn’t like being on her tummy, I put one ball here, another there, and then she didn’t cry—she stretched out to reach the paper. Once she got it, she looked at me and I said, “Very good!” She doesn’t crawl—my older son didn’t crawl either, he just went straight to walking—but I try to get her to use her hands and legs more.

Then we tried paper and paint, but she wanted to eat it. So my husband suggested trying fruit instead. He took her hand and said, “Look, (baby’s name)’s hand,” and so on. We also tried with orange and strawberry.

As for the books, she tried to eat them too. I said, “No, this is a book, look, let’s count.” One had a little bird—I’d show her and say, “Look, it was flying like this,” and I’d move her hand to show flying. Her brother would join in: “Look, sister, like this!” And he’d say, “What else, mummy?” And I’d add more: “There was a wolf…” I didn’t actually read the book—I made it up. And they’d go “Woooow!” And that’s when she started saying “Mummy, mummy.” Before, she only said “water.” Now she talks much more.

With the toys, we stuck down the tape and I said, “Let’s do a parade, let’s see.” I played it like it was something big—I don’t see her as small. We put the giraffe and all the animals, and I didn’t make the sounds myself—I played them on my phone and said, “See how it goes?” And she’d laugh. Every time the animal made a sound, she’d laugh. Then she’d take them and put them in her mouth, and every time she did, I’d play the sound again. It got her attention.

Then we had a little ball that made noise—it spun and spun, and she’d watch it from side to side. She does everything while dancing because we always have music on—my husband’s music from India—and she dances to it. My son and I joined her.

This wasn’t in the book or the handouts, but I made a bottle with water and put a clip shaped like a fish in it, so when she squeezed it, the fish would go up. Then I added a pen and two bottle caps with a string, and when she pulled it, it looked like a little car—it rolled. And she laughed out loud.

As for the fabric, we played hide-and-seek with it. Later, we didn’t even need the fabric—we’d just hide somewhere else and she’d look for us. Her brother played with her too.

The books were really interesting—so interactive—and it’s like they awakened something in her. She wasn’t very stable sitting before, but now she tries to grab the animals, reach for things with her hands, and the colours really catch her attention. It helped her a lot—now she can sit much better.

**Researcher:** That’s amazing, how you adapted and expanded! Was that something you used to do with your son?
**Mum:** No, that all came from this. I didn’t do any of that with my son. But it’s something about me—I always try to go the extra mile. If they tell me to just change a battery at work, I look and see what else might be needed—I always try to do more than what’s asked. So I read, looked, and said, “Oh, I can do this too.” For example, I took medicine bottles—like ibuprofen—washed them and filled them with lentils, little stones, and each made a different sound. Then I watched to see which sound she liked best. And with music, she picks her favourite and starts moving and dancing.

It fills me with joy to see that I offer her activities and she wants more. Then she naps just 15 minutes and wakes up ready to do another activity.

**Director**
Two teachers want to share their experience with the Oxford boxes. They sat down to play with the boxes, and I told them—this was the starting point. It sparks your imagination—you can soar. This is just the beginning—we don’t know where it might lead. One of them picked a child from her group to try it with. She’s going to tell us about her wonderful experience, where the child, just by seeing the materials, did more—and from there, it kept going...

**Teacher 1 (2-3 year olds)**
We asked in class who wanted to go do a special activity, and several hands went up. So I took them all, even some two-year-olds. I sat down to play and invited them to see what might happen. I read the books, made the little house—all of it. I set up an activity at the table, and they loved it. But then they kept asking for more—that’s what happens when something captures their interest. That’s why it’s great to allow for open-ended engagement with these materials, because it opens up so many possibilities.

There was one particular game, the memory game, and I would highlight things, take one out, then another. Then they started asking to organise it themselves, to take it out, arrange it a certain way, mix it with the blanket and put it back. From there, many activities and patterns emerged—ones that they proposed themselves. I was open to what they suggested. It was very enriching because when we returned to the classroom, they told the others about it: “Did you see the game...?” And the rest asked me for it. I wanted to ask if I could borrow the box for the other 16 kids—they want to try it too.

We also used it with the two-year-olds—we did the book activity, the one with animals. They started naming them, making the sounds, naming the colours—it opened up so many opportunities. It was very enriching and lovely with the kids. So, we’re more than grateful for the opportunities. It helps us too—it gives us ideas and encourages us to incorporate new activities. It’s great to open up more. It helped me get more ideas and let the children lead the play. That’s what makes it special—you can suggest something, but if they build on it, challenge themselves, it’s much richer for them and for us as observers.

**Teacher 2 (1-year-olds)**
Here in our class, we have kids from 8 months to 2 years. We also invited the kids to try out the activities. One little girl in particular—we offered her the game with the coloured ribbons and animals. At first, she noticed something was going on, but didn’t connect it with the animal. Then she looked at me, started naming the animals and making the sounds. She even started stacking them on top of each other—so there was a whole new proposal.

Later in the classroom, we explored the folding book—it was a morning activity when they’re calmer. That book led us to a song, to making sounds. And if they didn’t know the sounds, they’d make them up. They’d start saying, “It has hands, it has feet—how many legs does it have?” Their imagination is so rich. This helped us a lot. We’re going to keep using the box—we’ll borrow it if we need to because it’s beautiful to see their imagination grow from something so simple. We let them be free and experiment, and they teach us too—we’re here to learn.

We also tried painting with tempera. We started with pencils, crayons, and now the kids paint their folders by themselves—we just let them express themselves. We’re very grateful and will keep using the box—it’s wonderful to watch them grow.

**Mum 6**
The truth is the box was really helpful. My child is one year and three months old, he really likes the paint and the crayons, he loves painting—but only on paper, not on the fabric. He likes to paint; he grabs his older brother’s notebooks and starts drawing all over them. He loved the little animals—there was a dinosaur that opens its mouth and makes sounds, and he loves it. There was also a little monkey and a lion. I ask him how the monkey goes, and he goes “oo oo oo.” He’s still working on the lion, but every day he tries to make the animal sounds. It helped him so much—he’s now saying a lot more, his language really took off. And now when you tell him to get something, he actually goes and gets it—he understands a lot more than before.

He’s also really drawn to the little book, to the colours and the pictures. After getting the box, I started reading stories to him—I never did that with his older brother. And now he brings me the book himself, the one with Ratón Gastón, he brings it over and shows it to me, and I tell him the story and point out who the characters are. I really enjoyed spending that time reading with him—it feels like I’m learning too, like I’m learning all over again.

And the little tent—we set it up with two stools and put the fabric over it, and he goes inside and puts everything in there—shoes, everything, he puts it all under the fabric. His brother plays with him too. He’s learning so much—honestly, we really enjoyed it. Thank you so much.

**Dad 7**
For me, it was also that sometimes you just run out of ideas for play, right? And this gave me more ideas—like with the fabric, the paints… (inaudible)… the only thing he doesn’t like is tidying up! But yes, it really helped me a lot, especially with ideas—like the one with the ribbons. It was honestly really good.

**Mum 8**
It really helped me a lot. I have four children, and I don’t remember ever doing any kind of activity with them. I don’t remember sitting down with them, giving them attention to play, or helping them. And now with [baby’s name]… her siblings actually complain that I never did any of this with them, and now I do it with her. When I got the box, I thought—this isn’t something to just put away like the director said. So I went and sat down with [baby’s name], just the two of us, and then one of her brothers came and asked, “What are you doing?” And I said, “We’re playing with [baby’s name], seeing what she does.” And of course, then came the complaints—and they’re right. I never paid that kind of attention to them, I never focused on their ideas or gave them the time they needed while growing up.

I don’t even know why, because back then I wasn’t working—I was with them all the time, but not playing with them or giving them focused time. And with [baby’s name] it’s helped me a lot—I can see she’s starting to talk when she looks at the pictures, she grabs a toy and brings it to you, she wants you to play with her. But the problem is, I have a five-year-old son who is *very* jealous. When I play with [baby’s name], he says, “You don’t love me. When you’re with her, I don’t exist.” And that really upsets me. I’ve been working for three years now, so I’m no longer at home all the time. The only time we really talk is at dinner. We sit and talk, but their dad wants to eat in peace and quiet. And I tell him—this is our moment to talk, to see how school or nursery went. Because after dinner it’s bedtime, and then I have to get up at 4:30.

My children think I spend all my time with [baby’s name], but they don’t know I drop her at nursery, go to work, pick her up, and come home. The box really helped me—it gave me the idea to sit down and do something more than just show love. Of course, I care about all my children, but it pushed me to actually *do* something with them, to spend more time together. The box is a great idea—it gave me so many ideas, and even if some things didn’t work, we used them in other ways to come up with new things. It really let me spend quality time with my daughter.

I just keep asking myself—why didn’t I do this with my older kids? I wasn’t working, I had the time. That was my mistake—I admit it. I just didn’t know. But it hurts now when they say, “Why didn’t you do this with me?” Now I’m trying to do something with each of them, to give them attention and be there for them.

**Mum 9**
The box helped us a lot. I had an accident, lost my memory—I lost a lot. And I really clung to my son. I became overprotective, and I kept limiting him—I didn’t want anything bad to happen to him. I was very unsure, very hesitant about doing things—I didn’t feel confident. Then we got the box, and everything changed. Now he wakes up at 6 a.m., calling “Mummy, mummy, it’s time!” He gets up ready to play.

Since we got the box, I’ve changed my expectations—and so has his dad. He used to hold her but didn’t really know what to do with her. Now he takes out the box, gets the little animals, and spends quality time with her—even if it’s just five or ten minutes, he gives her that time. That’s a big relief for us, too—it gives me a bit of time to do other things. I really think this is *so* important—so fathers are part of parenting too. Often, women carry all the weight because the men don’t get involved.

I also include her older brother—he has different ideas, and we try to make him part of it too. My daughter is really happy. The box was genuinely great—it gave me lots of ideas for helping her grow. It made me feel more confident. Honestly, it was a huge help for me as a mum.

**Dad 10**
For me, the box helped a lot with organising all the activities in one place. We already had a lot of toys for him—we keep them within reach so he can help himself. The box we got had marine animals, so we included them during bath time. The whale, for example, was big enough to “eat” the other fish. He played with the seal… he really liked it.

He also played a lot with the fabric, used his free time to draw. He prefers open spaces, so he’d go out onto the balcony—and he loved getting messy, and I think that’s a great stage for children to enjoy that. We also taught him that all the activities belonged in the box, so he had to figure out how to fit everything back in—he always left it all tidy in the end.

He really likes trying new things—like all kids—so after a while he’d come up with new ways to use the same toys. We also included his two older siblings—they always got involved and helped him make the animal sounds. As for the book, he really enjoyed the images—I think it helped him focus better and be more coordinated in what he wanted to do.

We found the whole idea very positive. We already made time for our children as part of our daily routine. For example, the older ones play Cluedo—he doesn’t know the rules yet, but he still wants to join in. We really liked the activity box—it gave us lots of new ideas.
