# Supplementary material for: Shaping Development Through Parents: A Scalable Approach to Early Learning
Source: Dev Sci. 2026 Sep 4;29(6):e70279. doi: 10.1111/desc.70279 (PMC13543324; doi:10.1111/desc.70279)
Supplement: Supplementary file 7 [file DESC-29-e70279-s007.docx]

**Supplementary Material 3**

*Parent Feedback Questionnaire*

| Original item in Spanish | English translation | Scale | Original answer options in Spanish | English translation |
| --- | --- | --- | --- | --- |
| ¿Qué edad tiene tu hijo(a)? | How old is your child? | NA | Pregunta abierta | Open question |
| ¿Qué tan seguido hiciste alguna de las actividades con tu hijo(a)? | How often did you do any of the activities with your child? | Five-point Likert scale | Nunca / Casi nunca / Ocasionalmente / Casi todos los días / Todos los días | Never / Rarely / Occasionally / Almost every day / Every day |
| El paquete de actividades me ha animado a pasar más tiempo realizando actividades con mi hijo. | The activity pack encouraged me to spend more time doing activities with my child. | Four-point Likert scale | Totalmente en desacuerdo / En desacuerdo / De acuerdo / Totalmente de acuerdo | Strongly disagree / Disagree / Agree / Strongly agree |
| El paquete de actividades ha ampliado la variedad de actividades que hago con mi hijo(a). | The activity pack expanded the variety of activities I do with my child. | Four-point Likert scale | Totalmente en desacuerdo / En desacuerdo / De acuerdo / Totalmente de acuerdo | Strongly disagree / Disagree / Agree / Strongly agree |
| El paquete de actividades ha aumentado mi confianza en apoyar el desarrollo de mi hijo(a). | The activity pack increased my confidence in supporting my child’s development. | Four-point Likert scale | Totalmente en desacuerdo / En desacuerdo / De acuerdo / Totalmente de acuerdo | Strongly disagree / Disagree / Agree / Strongly agree |
| Como resultado de este paquete de actividades, he notado mejoras en el desarrollo de mi hijo(a). | As a result of this activity pack, I have noticed improvements in my child’s development. | Four-point Likert scale | Totalmente en desacuerdo / En desacuerdo / De acuerdo / Totalmente de acuerdo | Strongly disagree / Disagree / Agree / Strongly agree |
| ¿El paquete de actividades ha hecho alguna diferencia en usted o en su hijo(a) de alguna otra manera? Si / No (de ser así, explique) | Has the activity pack made any other difference for you or your child? Yes / No (if so, please explain) | NA | Si/No + Pregunta abierta | Yes/No + Open question |
| Disfruté hacer las actividades con mi hijo(a) | I enjoyed doing the activities with my child. | Four-point Likert scale | Totalmente en desacuerdo / En desacuerdo / De acuerdo / Totalmente de acuerdo | Strongly disagree / Disagree / Agree / Strongly agree |
| A mi hijo(a) le gustaron las actividades | My child enjoyed the activities. | Four-point Likert scale | Totalmente en desacuerdo / En desacuerdo / De acuerdo / Totalmente de acuerdo | Strongly disagree / Disagree / Agree / Strongly agree |
| ¿Tienes alguna sugerencia sobre cómo podemos mejorar estos paquetes de actividades? | Do you have any suggestions on how we can improve these activity packs? | NA | Pregunta abierta | Open question |
| ¿Hay algo más que te gustaría compartir con nosotros? | Is there anything else you would like to share with us? | NA | Pregunta abierta | Open question |
