# Supplementary material for: Shaping Development Through Parents: A Scalable Approach to Early Learning
Source: Dev Sci. 2026 Sep 4;29(6):e70279. doi: 10.1111/desc.70279 (PMC13543324; doi:10.1111/desc.70279)
Supplement: Supplementary file 8 [file DESC-29-e70279-s008.docx]

**Supplementary Material 7**

*Example Codes and Generated Themes and Subthemes* *from Parent and Centre Staff Discussions and Interviews*

| Example Codes | Themes | *Subthemes* |
| --- | --- | --- |
| Child loved messy play | **A Children’s enjoyment** |  |
| Packs made children want more |  |  |
| Child enjoyed books |  |  |
|  |  |  |
| Activities got older sibling to play with child too | **B Stronger family connections** |  |
| Parent or teacher notices details about child |  |  |
| Pack brought family closer together |  |  |
|  |  |  |
| Books encouraged child’s imagination | **C Developmental gains** |  |
| Child’s attention has improved |  |  |
| Painting and markers helped child develop |  |  |
|  |  |  |
| Pack helped parent learn to play with child | **D Support for caregivers** | *D.1 Upskilling parents* |
| Pack freed teacher/parent/family members to be creative |  | *D.2 Empowering parents and teachers* |
| Pack helped mum by involving family in playing with child |  | *D.3 Practical and emotional benefits for mothers* |
|  |  |  |
| More paints would be good | **E – Future improvements** |  |
| Baby put everything in mouth at first |  |  |
