# Supplementary material for: Shaping Development Through Parents: A Scalable Approach to Early Learning
Source: Dev Sci. 2026 Sep 4;29(6):e70279. doi: 10.1111/desc.70279 (PMC13543324; doi:10.1111/desc.70279)
Supplement: Supplementary file 9 [file DESC-29-e70279-s009.docx]

**Supplementary Material 2**

The Social Vulnerability Index (SVI) is constructed by summing the number of vulnerability indicators present in each census sector. The more indicators present, the higher the area's vulnerability. These indicators may include:

Poverty: Percentage of households or individuals in poverty, including income poverty and multidimensional poverty.

Extreme Poverty: Percentage of households or individuals in extreme poverty.

Homelessness: Number of people living on the streets.

Access to Services: Difficulties accessing basic services such as water, sanitation, electricity, etc.

Housing Conditions: Quality of housing and overcrowding.

Health: Infant mortality rates, prevalent diseases, etc.

Education: Educational attainment, school dropout rates, etc.

Employment: Unemployment, underemployment, informal labor, etc.

Demographic Factors: Age, family composition, etc.
