# Supplementary material for: Shaping Development Through Parents: A Scalable Approach to Early Learning
Source: Dev Sci. 2026 Sep 4;29(6):e70279. doi: 10.1111/desc.70279 (PMC13543324; doi:10.1111/desc.70279)
Supplement: Supplementary file 10 [file DESC-29-e70279-s010.docx]

**Supplementary Material 9**

Bayley’s Language Difference Score Q-Q Plot
